# Tau burden and the functional connectome in Alzheimer’s disease and progressive supranuclear palsy

**DOI:** 10.1093/brain/awx347

**Published:** 2018-01-05

**Authors:** Thomas E Cope, Timothy Rittman, Robin J Borchert, P Simon Jones, Deniz Vatansever, Kieren Allinson, Luca Passamonti, Patricia Vazquez Rodriguez, W Richard Bevan-Jones, John T O'Brien, James B Rowe

**Affiliations:** 1 Department of Clinical Neurosciences, University of Cambridge, Cambridge, UK; 2 Department of Psychology, University of York, York, UK; 3 Division of Anaesthesia, Department of Medicine, University of Cambridge, Cambridge, UK; 4 Department of Psychiatry, University of Cambridge, Cambridge, UK; 5 Department of Pathology, Cambridge University Hospitals NHS Foundation Trust, Cambridge, UK; 6 Medical Research Council Cognition and Brain Sciences Unit, Cambridge, UK

**Keywords:** Alzheimer’s disease, progressive supranuclear palsy, tau, functional connectivity, graph theory

## Abstract

Alzheimer’s disease and progressive supranuclear palsy (PSP) represent neurodegenerative tauopathies with predominantly cortical versus subcortical disease burden. In Alzheimer’s disease, neuropathology and atrophy preferentially affect ‘hub’ brain regions that are densely connected. It was unclear whether hubs are differentially affected by neurodegeneration because they are more likely to receive pathological proteins that propagate trans-neuronally, in a prion-like manner, or whether they are selectively vulnerable due to a lack of local trophic factors, higher metabolic demands, or differential gene expression. We assessed the relationship between tau burden and brain functional connectivity, by combining *in vivo* PET imaging using the ligand AV-1451, and graph theoretic measures of resting state functional MRI in 17 patients with Alzheimer’s disease, 17 patients with PSP, and 12 controls. Strongly connected nodes displayed more tau pathology in Alzheimer’s disease, independently of intrinsic connectivity network, validating the predictions of theories of trans-neuronal spread but not supporting a role for metabolic demands or deficient trophic support in tau accumulation. This was not a compensatory phenomenon, as the functional consequence of increasing tau burden in Alzheimer’s disease was a progressive weakening of the connectivity of these same nodes, reducing weighted degree and local efficiency and resulting in weaker ‘small-world’ properties. Conversely, in PSP, unlike in Alzheimer’s disease, those nodes that accrued pathological tau were those that displayed graph metric properties associated with increased metabolic demand and a lack of trophic support rather than strong functional connectivity. Together, these findings go some way towards explaining why Alzheimer’s disease affects large scale connectivity networks throughout cortex while neuropathology in PSP is concentrated in a small number of subcortical structures. Further, we demonstrate that in PSP increasing tau burden in midbrain and deep nuclei was associated with strengthened cortico-cortical functional connectivity. Disrupted cortico-subcortical and cortico-brainstem interactions meant that information transfer took less direct paths, passing through a larger number of cortical nodes, reducing closeness centrality and eigenvector centrality in PSP, while increasing weighted degree, clustering, betweenness centrality and local efficiency. Our results have wide-ranging implications, from the validation of models of tau trafficking in humans to understanding the relationship between regional tau burden and brain functional reorganization.

## Teaser

Prion-like, trans-neuronal spread of tau pathology in humans is controversial. By evaluating tau burden and functional connectivity in living patients, Cope *et al.* demonstrate relationships consistent with this in Alzheimer's disease but not progressive supranuclear palsy. Tau distribution in the latter is better explained by metabolic demand and trophic support.

## Introduction

Alzheimer’s disease and progressive supranuclear palsy (PSP) are both characterized by intracellular neurofibrillary lesions containing hyper-phosphorylated filamentous tau inclusions (Goedert and Spillantini, 2006). However, the diseases differ in: (i) the distribution of these tau inclusions; (ii) the balance of expression of tau isoforms; and (iii) the ultrastructure of tau filaments. Here, we test the impact of these differences in tau pathology on the reorganization of large-scale functional brain connectivity architecture.

Alzheimer’s disease is characterized by widespread extracellular deposition of amyloid-β and paired helical filaments of tau with three (3R) and four (4R) repeats in the microtubule-binding domain (Sisodia *et al.*, 1990; Liu *et al.*, 2001). In Alzheimer’s disease, these pathological proteins arise early in the transentorhinal cortex, from where they spread to limbic regions, followed by inferior frontal and parietal cortex (Braak and Braak, 1991, 1995). Although tau and amyloid-β have complex and synergistic effects (Nisbet *et al.*, 2015), there is converging evidence that tau mediates direct toxic effects on neurons and synaptic plasticity (Ballatore *et al.*, 2007; Roberson *et al.*, 2007; Ittner and Götz, 2011; Myeku *et al.*, 2016) and correlates with hypometabolism and symptomatology in Alzheimer’s disease (Lehmann *et al.*, 2013; Ossenkoppele *et al.*, 2015, 2016). By contrast, pathological tau deposits in PSP are composed of straight filaments of predominantly 4R tau (Taniguchi-Watanabe *et al.*, 2016). This is most prominent in midbrain and deep brain nuclei early in the course of the Richardson’s syndrome variant of PSP, spreading to cortical regions in advanced stages of the disease (Williams *et al.*, 2007).

To estimate the burden of tau pathology *in vivo*, PET ligands have been developed, including ^18^F-AV-1451 (Chien *et al.*, 2013; Xia *et al.*, 2013; Ossenkoppele *et al.*, 2015, 2016). While there has been significant debate about the specificity of this ligand for tau, especially in the non-Alzheimer’s dementias (Bevan-Jones *et al.*, 2017*a*; Xia and Dickerson, 2017), the distribution of ^18^F-AV-1451 binding recapitulates Braak staging in Alzheimer’s disease (Schwarz *et al.*, 2016) and correlates with post-mortem neuropathology in primary tauopathies (Smith *et al.*, 2016; Passamonti *et al.*, 2017). ^18^F-AV-1451 binding has been shown to correlate with cognitive performance in Alzheimer’s disease (Johnson *et al.*, 2016) and healthy older adults (Schöll *et al.*, 2016) more robustly than quantitative amyloid-β imaging (Brier *et al.*, 2016). The ^18^F-AV-1451 ligand also effectively distinguishes between Alzheimer’s disease and PSP cases based on both the intensity and regional distribution of its binding potential (BP_ND_) (Passamonti *et al.*, 2017).

In many neurodegenerative disorders, neuropathology and atrophy are most marked in those brain regions that are densely connected, both at the structural (Crossley *et al.*, 2014) and functional level (Dai *et al.*, 2014). In graph-theoretical terms, these densely connected regions are usually referred to as ‘hubs’ (Buckner *et al.*, 2009). There are a number of hypotheses as to why hubs are vulnerable to neurodegeneration. First, pathological proteins may propagate trans-neuronally, in a prion-like manner (Prusiner, 1984; Baker *et al.*, 1994; Goedert, 2015) such that highly connected regions are more likely to receive pathology from ‘seed’ regions affected in early stages of the disease (Zhou *et al.*, 2012), leading to neurodegeneration that mirrors structural and functional brain connectivity (Raj *et al.*, 2012, 2015; Abdelnour *et al.*, 2014). Alternatively, hubs might be selectively vulnerable to a given level of pathology, due to a lack of local trophic factors (Appel, 1981), higher metabolic demands (Saxena and Caroni, 2011; de Haan *et al.*, 2012), or differential gene expression (Rittman *et al.*, 2016).

These alternate hypotheses lead to different predictions about the relationship between tau burden and connectivity. The trans-neuronal spread hypothesis predicts that regions that are more strongly interconnected would accrue more tau pathology. This would manifest as higher tau burden in nodes with larger weighted degree, which is a measure of the number and strength of functional connections involving each node. In contrast, if hubs are vulnerable to tau accumulation because of increased metabolic demand this might manifest as a positive relationship between tau burden and participation coefficient, which is a measure of the proportion of a node’s connections that are with other neural communities, and is the graph metric that is most closely correlated with metabolic activity (Chennu *et al.*, 2017). Finally, if trophic support is an important factor in tau accumulation, this might manifest as a negative relationship between tau burden and clustering coefficient; nodes with less tightly clustered connectivity patterns might have more vulnerable trophic supply.

Here we go beyond previous associative studies to examine, in the same subjects, the relationship between *in vivo* tau burden, as measured by the PET ligand ^18^F-AV-1451 BP_ND_, and functional connectivity, as summarized by graph theoretic measures based on resting state (task-free) functional MRI. We test the following linked hypotheses:

- Brain regions that are normally more densely interconnected accrue more tau pathology.
- In Alzheimer’s disease, where tau accumulation is predominantly cortical, the functional consequence is that affected nodes become more weakly connected and local efficiency of information transfer is reduced.
- In PSP-Richardson’s syndrome, where neurodegeneration associated with tau accumulation is most severe in midbrain and basal ganglia, the functional consequence of disrupted cortico-subcortical and cortico-brainstem interactions (Gardner *et al.*, 2013) is that indirect cortico-cortical connections become stronger.

## Materials and methods

### Participants

All patients had mental capacity to take part in the study and provided informed consent. Study procedures were approved by the National Research Ethics Service. We recruited 17 patients with Alzheimer’s disease, as evidenced by a either clinical diagnosis of probable Alzheimer’s dementia according to consensus criteria (*n* = 10) (McKhann *et al.*, 2011), or a clinical diagnosis of mild cognitive impairment (MCI) and a positive amyloid PET scan (*n* = 7) (Klunk *et al.*, 2004; Okello *et al.*, 2009). Patients with MCI and evidence of amyloid-β were included to ensure the largest possible variability in tau burden within the Alzheimer’s disease group. We also recruited 17 patients with PSP-Richardson’s syndrome by 1996 criteria (Litvan *et al.*, 1996). Retrospective case review confirmed that all PSP subjects also met the revised 2017 criteria for PSP-RS (Höglinger *et al.*, 2017). Twelve age-matched controls were also examined. Participant demographics are given in Table 1 and detailed neuropsychological test results for each subject are shown in Supplementary Table 1.

**Table 1:** Participant demographics

Group	Number	Age, years	Years of education	MMSE	ACE-R	PSP-RS
Alzheimer’s disease	17	71 (9)	14 (3)	25 (4)	72 (14)	-
PSP	17	69 (6)	12 (2)	27 (4)	83 (14)	41 (14)
Controls	12	67 (8)	16 (2)	29 (1)	96 (3)	-

Values are mean (SD). ACE-R = Addenbrooke’s Cognitive Examination, Revised Edition; MMSE = Mini-Mental State Examination; PSP-RS = Progressive Supranuclear Palsy Rating Scale.

### Study procedures

This study formed part of the NIMROD (Neuroimaging of Inflammation in Memory and Related Other Disorders) project, for which the trial protocol containing general methods has been previously published (Bevan-Jones *et al.*, 2017*b*).

All participants underwent the Addenbrooke’s Cognitive Examination-Revised (ACE-R), 11 min of resting state functional MRI at 3 T, and ^18^F-AV-1451 PET imaging. Participants with PSP were also examined according to the PSP Rating Scale (Golbe and Ohman-Strickland, 2007). Those with a clinical diagnosis of MCI had ^11^C-PiB PET imaging on a separate occasion—only those with increased uptake indicative of underlying Alzheimer’s pathology are reported here.

### MRI data acquisition and preprocessing

MRI was performed at the Wolfson Brain Imaging Centre, University of Cambridge, UK using a 3 T Siemens Magnetom Tim Trio scanner with a Siemens 32-channel phased-array head coil (Siemens Healthcare).

A T_1_-weighted magnetization-prepared rapid gradient-echo (MPRAGE) image was acquired with repetition time = 2300 ms, echo time = 2.98 ms, matrix = 256 × 240, in-plane resolution of 1 × 1 mm, 176 slices of 1 mm thickness, inversion time = 900 ms and flip angle = 9°.

Eyes-closed resting state (task-free) multi-echo functional imaging was carried out for 11 min. A total of 269 EPI image volumes were acquired with repetition time = 2430 ms, echo times = 13.00, 30.55 and 48.10 ms, matrix = 64 × 64, in-plane resolution of 3.75 × 3.75 mm, 34 slices of 3.8 mm thickness with an interslice gap of 0.38 mm, GRAPPA parallel imaging with an acceleration factor of 2 and bandwidth = 2368 Hz/pixel. The first six volumes were discarded to eliminate saturation effects and achieve steady state magnetization. Preprocessing employed the ME-ICA pipeline (https://wiki.cam.ac.uk/bmuwiki/MEICA) (Kundu *et al.*, 2012, 2013), which uses independent component analysis to classify BOLD and non-BOLD signals based on the identification of linearly dependent and independent echo time dependent components. This provides an optimal approach to correct for movement-related and non-neuronal signals, and is therefore particularly well suited to our study, in which systematic differences in movement or head position might reasonably have been expected between patient groups. In fact, perhaps surprisingly, such differences were not observed (Supplementary Fig. 1)—between group ANOVAs demonstrated no significant differences between groups in terms of frame displacement before (*P* = 0.88) or after (*P* = 0.42) ME-ICA preprocessing, nor in terms of DVARS (Power *et al.*, 2012) before (*P* = 0.89) or after (*P* = 0.67) ME-ICA preprocessing. Note that both movement parameters were approximately an order of magnitude lower after ME-ICA preprocessing.

The MPRAGE images were processed into the standard space using DARTEL (Ashburner, 2007), producing a study-specific template in stereotactic space. Each functional MRI series mean image was co-registered to the corresponding MPRAGE image. The whole functional MRI series was warped to the template space using the DARTEL flow fields.

To perform a whole brain graph-theoretical analysis that included the brainstem, cerebellum and subcortical structures, the Harvard-Oxford Cortical atlas and Harvard-Oxford Subcortical atlas, each thresholded at 25%, were combined. Additionally, a Freesurfer 6 brainstem parcellation of the MNI152 (2009 asymmetric) brain together with remaining Ventral DC completed the whole brain labelling. This atlas was sub-parcellated into 598 regions of approximately equal volume [mean 1.995, standard deviation (SD) 0.323 ml] such that each sub-parcel could be uniquely identified with an atlas region. The MNI-space parcellation was matched to the group standard space using inverse deformations following application of the ‘Population to ICBM Registration’ SPM function to the group template, with nearest neighbour interpolation.

The blood oxygen level-dependent (BOLD) time series for each node was extracted using the CONN functional connectivity toolbox (Whitfield-Gabrieli and Nieto-Castanon, 2012). Between-node association matrices were generated, and then z-transformed for further analysis.

### Functional connectivity assessment

Graph theoretical analysis was used to investigate the global and local characteristics of brain networks. These metrics were calculated in python using the Maybrain software (github.com/rittman/maybrain) and networkx (version 1.11). Reported metrics were calculated in each subject at absolute network density thresholds of 1–10% in 1% increments using a minimum spanning tree to ensure complete connectivity of the graph (Alexander-Bloch *et al.*, 2010). Primary statistical analysis was performed at an intermediate density of 6%, with confirmatory analyses separately performed at all other densities. Weighted degree and participation coefficient were analysed in their raw forms, and all other metrics were dissociated from variation in degree by binarization after thresholding and normalization against 1000 random graphs with the same number of connections at each node.

The graph metrics assessed were:

- Weighted degree: the number and strength of functional connections involving each node.
- Weighted participation coefficient: the proportion of a node’s functional connectivity that involves other nodes that are not part of its own community structure, as defined by the Louvain community detection algorithm. Nodes with high degree and low participation coefficient are ‘provincial hubs’ (i.e. display strong connectivity only within their own community), while those with high participation coefficient are connector nodes (Joyce *et al.*, 2010).
- Betweenness centrality: the number of shortest paths between any other two nodes that pass through the node of interest. Nodes that are important for the transfer of information between other nodes have high betweenness centrality.
- Closeness centrality: the inverse of the path length between a node and all other nodes in the graph. This is the node-wise equivalent of global efficiency, which is the inverse sum of all the shortest path lengths in the graph.
- Local efficiency: the number of strong connections a node has with its neighbouring nodes. This reflects the robustness of local networks to disruption.
- Eigenvector centrality: this measure quantifies the functional influence of a node on every other node in the graph, by weighting the importance of each nodal connection based on the influence of the nodes with which they connect.
- Clustering coefficient: the fraction of triangular connections formed by a node with other nodes. In other words, a node is strongly clustered if a large proportion of its neighbours are neighbours of each other.

Nodal connectivity strength was assessed for comparison to weighted degree, to ensure that our results did not result from bias introduced by proportionate thresholding. This metric is related to weighted degree, but includes information from all strengths of connection between every pair of nodes. As such, it is more subject to functional MRI signal-to-noise ratio limitations, and it is not a suitable metric for whole-brain, cross-sectional analysis across individuals, but it can be used to make a node-wise, group average assessment analogous to that for weighted degree.

### Tau burden assessment

Other than the atlas used to define regions of interest, all AV-1451 and PiB data acquisition and preprocessing steps were identical to those reported by Passamonti *et al.* (2017). Ligand preparation was carried out at the Wolfson Brain Imaging Centre, University of Cambridge, with high radiochemical purity. ^18^F-AV-1451 was produced with a specific activity of 216 ± 60 GBq/μmol at the end of synthesis (60 min); ^11^C-PiB specific activity was >150 GBq/μmol. PET imaging was performed on a GE Advance PET scanner with a 15 min ^68^Ge transmission scan used for attenuation correction acquiring 58 frames of increasing duration. PET scanning was performed in 3D mode (63 image planes; 15.2 cm axial field of view; 5.6 mm transaxial resolution and 2.4 mm slice interval) 80–100 min after a 9.0 to 11.0 mCi bolus injection in frames of 4 × 5 min. Each ^11^C-PiB scan was acquired using a 8.5 to 15 mCi bolus injection immediately followed by a 60-min dynamic acquisition in 69 frames (12 × 15 s, 57 × 60 s). Scans were reconstructed on the GE Advance scanner using the PROMIS 3D filtered back projection algorithm, correcting for randoms, dead time, normalization, sensitivity and scatter attenuation.

The cerebellar grey matter was used as a reference tissue to express the distribution volume ratio (DVR) for the ^11^C-PiB PET data for each MCI participant. PiB scans were classified as positive if the average SUVR values across the cortex were more than 1.5 times that of the cerebellar regions of interest. Seven MCI participants met this criterion and were included in the study.

The non-displaceable binding potential of ^18^F-AV-1451 was assessed at each region of interest in the sub-parcellated atlas after rigid registration of each subject’s dynamic PET image series to their T_1_-weighted MRI scan. This was normalized against the superior cerebellum, a reference region considered to have no tau pathology in either PSP or Alzheimer’s disease (Williams *et al.*, 2007; Schöll *et al.*, 2016; Passamonti *et al.*, 2017). These data were corrected for white matter and CSF partial volumes by calculating the ordinary least squared solution for the binding potential map voxel-wise in each region for grey plus white matter segments, each smoothed to PET resolution.

### Statistical approach

A stereotyped statistical approach was taken to the analysis of graph metrics of theoretical interest. All statistical analyses were performed in MATLAB 2015b (The Mathworks Inc., 2015), with the exception of moderation analysis, which was performed in R (R Core Team, 2016).

A group-averaged analysis assessed the first hypothesis by examining the node-wise relationships between ^18^F-AV-1451 binding potential and weighted degree, weighted participation coefficient, and clustering coefficient. These analyses were performed across the whole brain and within 10 intrinsic connectivity networks. To avoid circularity, where the regions of interest are defined by the test data, the intrinsic connectivity networks were independently derived from a publicly available dataset (Smith *et al.*, 2009). A binary mask was constructed for each resting state network by thresholding at Z ≥ 2.6 (*P* < 0.005 uncorrected). Nodes were defined as belonging to a network if their centre of mass was within 5 mm of any positive voxel.

A between-subject analysis assessed the second and third hypotheses. This was undertaken at a variety of spatial scales. We first looked across the whole brain. For each individual, we first calculated a measure of disease-related tau burden. For participants with Alzheimer’s disease, in whom tau deposition increases in both magnitude and distribution as disease progresses (Braak and Braak, 1995), tau burden was calculated as average ^18^F-AV-1451 binding potential across the whole brain. In PSP tau deposition remains confined to brainstem and deep nuclei even in late disease (Williams *et al.*, 2007); tau burden was therefore calculated as average ^18^F-AV-1451 binding potential across the left and right thalamus, caudate, putamen, pallidum, accumbens, ventral diencephalon, midbrain, pons and medulla. Both methods were separately assessed for controls. These measures of individual tau burden were then correlated with whole-brain averaged graph metrics to assess the relationship between the metric in question and disease burden in each group separately. Moderation analysis was performed for each metric to assess whether the relationships differed between Alzheimer’s disease and PSP. It has recently been reported that patients with early onset Alzheimer’s disease have a higher tau burden than those with later onset, especially in later Braak stage regions (Schöll *et al.*, 2017). Non-significant trend relationships between overall tau burden and age were observed in our cohort (Alzheimer’s disease r = −0.27, PSP r = −0.02, Control r = −0.38; Supplementary Fig. 2). Cross-sectional analyses were therefore performed twice, with and without partialling out the effect of age on tau burden.

Regionality of the demonstrated effects was assessed within groups by correlating tau burden with the single subject graph metric values at each node; the gradient of the best fit linear regression within each group was the outcome measure. The nodal gradients in cerebellar regions were discarded (as this was the reference region for PET imaging), and the remaining regional maps were then collapsed into a vector. This was correlated with a matching vector of local tau burden at each node, calculated as the group-averaged increase in ^18^F-AV-1451 binding potential. This resulted in a measure of the relationship between the distribution of disease-related change in each graph metric and regional deposition of tau.

## Results

### Resting state connectivity differences exist between groups

**Figure 1:**
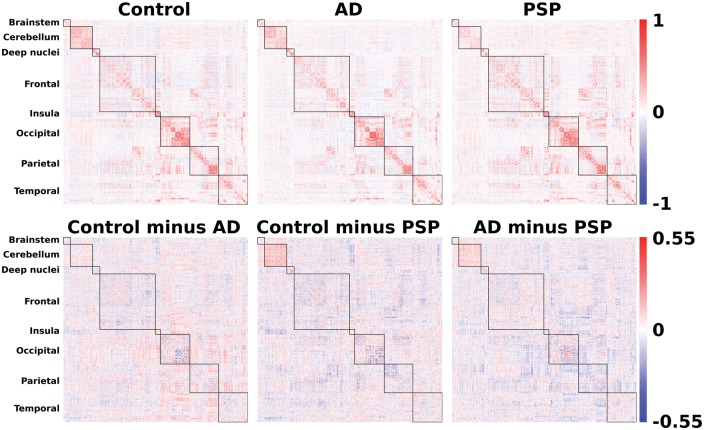
**Connectivity matrices.** *Top*: Association matrices derived from resting state (task-free) functional MRI. *Bottom*: Pairwise subtractions of the Fisher-z transformed association matrices to illustrate the large scale within and between-region disease-related changes in functional connectivity. AD = Alzheimer’s disease.

As a prelude to the detailed analysis of network features, we first confirmed that our groups differed in their resting state connectivity, by testing for differences between the group averaged association matrices (Fig. 1, top). Jennrich tests confirmed highly significant statistical differences between controls and Alzheimer’s disease (χ^2^ = 1.15 × 10^8^, *n*1 = 12, *n*2 = 17, *P* < 0.0001); controls and PSP (χ^2^ = 975 547, *n*1 = 12, *n*2 = 17, *P* < 0.0001); and Alzheimer’s disease and PSP (χ^2^ = 733 030, *n*1 = 17, *n*2 = 17, *P* < 0.0001).

The nature of these differences would be opaque without abstraction techniques. Pairwise subtraction of the Fisher transformed association matrices (Fig. 1, bottom) revealed the presence of structure within the data, with both within-lobe and between-lobe group differences. While one can make general observations from these raw data, such as a prominent reduction in cerebello-cerebellar and brainstem-cerebellar connectivity in PSP, and a general reduction in between-region connectivity in Alzheimer’s disease, the insights would be limited. Instead, graph theoretic measures allow one to examine which specific properties of the network underpin disease-related differences. For this approach each region in the brain becomes a node in a graph, which is functionally connected to all other nodes with a finite strength that is given by their pairwise correlation over time. Each individual’s graph is then thresholded so that the highest ‘*x*’ percentage of connections survive. Effects are sought that are consistently present at a variety of network thresholds. Here we examine network thresholds from 1 < *x* < 10%, representing a range of graphs from sparse to dense. Very sparse graphs contain less information and can miss important relationships. Conversely, very dense graphs are more subject to noise and, when binarized, begin to provide less meaningful information. Therefore, in what follows, we present the primary statistical analyses at an intermediate density of 6%, with statistical detail given for this density. To assess the robustness of effects to different thresholding decisions we note separately the range of network densities exhibiting statistical significance.

### Findings in Alzheimer’s disease but not PSP are consistent with trans-neuronal spread of tau

**Figure 2:**
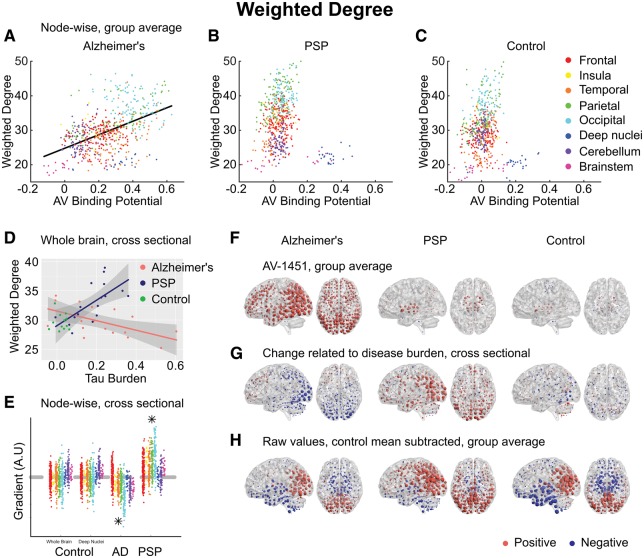
**Weighted degree**. (**A–C**) Group-averaged connection strength at each node, quantified by weighted degree, plotted against ^18^F-AV-1451 binding potential at that node. A statistically significant linear relationship was demonstrated only in Alzheimer’s disease (AD) (Pearson’s r = 0.48, *P* < 0.0001, Spearman’s rho = 0.48, *P* < 0.0001), and the corresponding regression line is plotted for this group. (**D**) Between-subjects analysis of the relationship between global tau burden and each weighted degree at a network density of 6%. No significant relationship was found for the control subjects with either method of assessing tau burden—for simplicity only their whole-brain average points are illustrated here. Moderation analysis for a differential relationship between graph metric and tau burden in the two disease groups (Alzheimer’s disease and PSP) was statistically significant. (**E**) The magnitude of disease-related change at each node is plotted as a single point, grouped by lobe. Control data are shown for both methods of assessing tau burden. Stars represent statistically significant excesses of positive or negative gradients in the disease groups. (**F**) Average ^18^F-AV-1451 binding potential at each node. Red spheres represent increases compared to the cerebellar reference region. Blue spheres represent decreases. The centre of the sphere is placed at the centre of the region of interest. The radius of each sphere is linearly related to the magnitude of binding at that node. All groups are scaled identically. (**G**) The local tau burden-related change in weighted degree is plotted for each group at each node. Red spheres represent local increases as a result of greater overall tau burden; blue spheres represent local decreases. The radius of each sphere is linearly related to the magnitude of disease-related change at that node across the whole range of disease burden observed in each group. All groups are scaled identically. Control images are based on the whole-brain method of assessing tau burden. (**H**) Average raw values for weighted degree within each group. The control mean for each metric is subtracted at every node. Red spheres represent increases compared to the control mean value. Blue spheres represent decreases. The centre of the sphere is placed at the centre of the region of interest. The radius of each sphere is linearly related to the difference from the control mean at that node. All groups are scaled identically.

We hypothesized that trans-neuronal spread of tau would manifest as a positive relationship between ^18^F-AV-1451 and weighted degree. As we have parcellated the brain into nodes of equal size, weighted degree is a measure of the volume of cortex to which a node is connected, and the strength of these connections. In Alzheimer’s disease, a strong positive correlation was observed at all network thresholds, such that the most strongly connected nodes had higher ^18^F-AV-1451 BP_ND_ (Pearson’s r = 0.48, *P* < 0.0001, Spearman’s rho = 0.48, *P* < 0.0001) (Fig. 2A). A consistent relationship was not present in PSP (Pearson’s r = −0.09, Spearman’s rho = 0.12) (Fig. 2B) or controls (Pearson’s r = 0.03, Spearman’s rho = 0.11) (Fig. 2C). These patterns were present at all examined network density thresholds.

**Figure 3:**
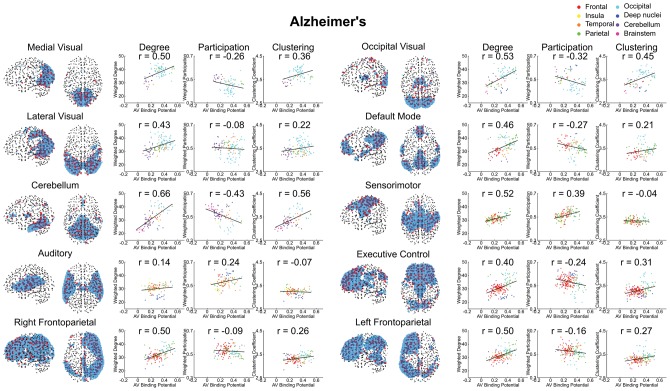
**Alzheimer’s disease comparison of the three graph metrics representing the three principal hypotheses of hub vulnerability.** Broken down by intrinsic connectivity network defined from Smith *et al.* (2009). The group-averaged graph metric at each node within a network is plotted against ^18^F-AV-1451 binding potential at that node. The Pearson correlation coefficient is noted in each case. Only weighted degree demonstrated a consistent relationship across all networks in keeping with its related hypothesis.

It is important to acknowledge that the degree of a node as defined by correlated activity is impacted by the size of the connectivity network to which it belongs (Power *et al.*, 2013). Therefore, one concern might be that correlations between ^18^F-AV-1451 and degree are driven by the coincidence that Alzheimer’s disease happens to affect the default mode network (DMN), which happens to be a large intrinsic connectivity network. To exclude this possibility, we undertook two additional analyses. First, we re-examined ^18^F-AV-1451 and degree when nodes belonging to the DMN were excluded; this did not abolish the strong relationship (Pearson’s r = 0.43, *P* < 0.0001). Second, we examined the relationship within each intrinsic connectivity network (Fig. 3). A strong positive relationship between ^18^F-AV-1451 and degree was seen in 9 of 10 networks examined (Pearson’s r ≥ 0.4, *P* < 0.0001). In contrast, no strong positive correlations observed in any network in PSP (Supplementary Fig. 3).

Finally, in each group we examined the relationship between each node’s unthresholded connectivity strength and ^18^F-AV-1451 BP_ND_ (Supplementary Fig. 4). This is a local measure of the strength of functional connectivity that does not rely on thresholding the graph and therefore takes into account both strong and weak connections. The pattern of results observed in weighted degree was replicated; in Alzheimer’s disease, a positive correlation was observed (r = 0.28, *P* < 0.0001), but no significant relationship was observed in PSP (r = −0.05, *P* = 0.22) or controls (r = 0.04, *P* = 0.31).

### Findings in PSP but not Alzheimer’s disease are consistent with selective vulnerability

**Figure 4:**
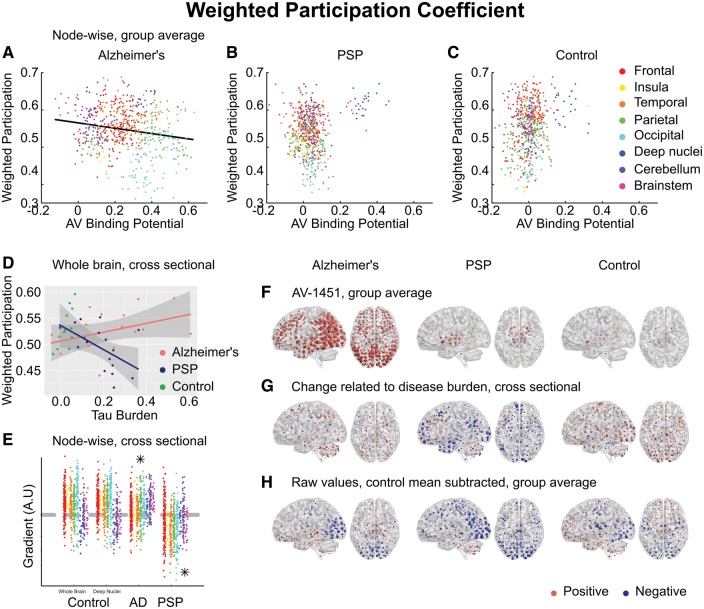
**Weighted participation coefficient.** (**A–C**) Group-averaged weighted participation coefficient at each node, plotted against ^18^F-AV-1451 binding potential at that node. A negative relationship was observed in Alzheimer’s disease (AD), in violation of the metabolic demand hypothesis. (**D**) Between-subjects analysis of the relationship between global tau burden and participation coefficient at a network density of 6%. No significant relationship was found for the control subjects with either method of assessing tau burden. Moderation analysis for a differential relationship between graph metric and tau burden in the two disease groups (Alzheimer’s disease and PSP) was statistically significant. (**E**) The magnitude of disease related change at each node is plotted as a single point, grouped by lobe. Control data are shown for both methods of assessing tau burden. Stars represent statistically significant excesses of positive or negative gradients in the disease groups. (**F**) Average ^18^F-AV-1451 binding potential at each node. Red spheres represent increases compared to the cerebellar reference region. Blue spheres represent decreases. The centre of the sphere is placed at the centre of the region of interest. The radius of each sphere is linearly related to the magnitude of binding at that node. All groups are scaled identically. (**G**) The local tau burden-related change in participation coefficient is plotted for each group at each node. Red spheres represent local increases as a result of greater overall tau burden; blue spheres represent local decreases. The radius of each sphere is linearly related to the magnitude of disease-related change at that node across the whole range of disease burden observed in each group. All groups are scaled identically. Control images are based on the whole-brain method of assessing tau burden. (**H**) Average raw values for participation coefficient within each group. The control mean for each metric is subtracted at every node. Red spheres represent increases compared to the control mean value. Blue spheres represent decreases. The centre of the sphere is placed at the centre of the region of interest. The radius of each sphere is linearly related to the difference from the control mean at that node. All groups are scaled identically.

We hypothesized that tau accumulation due to metabolic demand would manifest as a positive relationship between ^18^F-AV-1451 and weighted participation coefficient. This was not observed in Alzheimer’s disease; in fact there was a weak negative correlation (Pearson’s r = −0.18) (Fig. 4A).

In PSP, however, those nodes that displayed elevated ^18^F-AV-1451 were those that had the highest participation coefficient (Fig. 4B).

**Figure 5:**
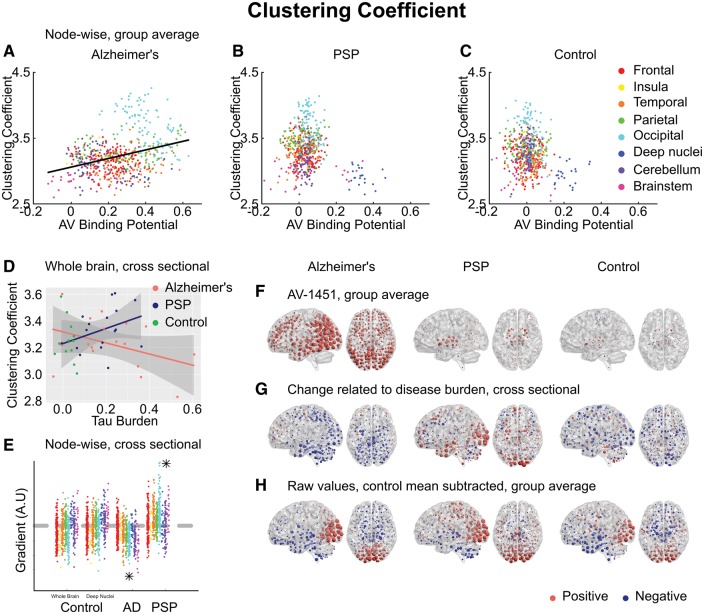
**Clustering coefficient.** (**A–C**) Group-averaged clustering coefficient at each node, plotted against ^18^F-AV-1451 binding potential at that node. A positive relationship was observed in Alzheimer’s disease (AD), in violation of the trophic support hypothesis. (**D**) Between-subjects analysis of the relationship between global tau burden and clustering coefficient at a network density of 6%. No significant relationship was found for the control subjects with either method of assessing tau burden. Moderation analysis for a differential relationship between graph metric and tau burden in the two disease groups (Alzheimer’s disease and PSP) was statistically significant. (**E**) The magnitude of disease related change at each node is plotted as a single point, grouped by lobe. Control data are shown for both methods of assessing tau burden. Stars represent statistically significant excesses of positive or negative gradients in the disease groups. (**F**) Average ^18^F-AV-1451 binding potential at each node. Red spheres represent increases compared to the cerebellar reference region. Blue spheres represent decreases. The centre of the sphere is placed at the centre of the region of interest. The radius of each sphere is linearly related to the magnitude of binding at that node. All groups are scaled identically. (**G**) The local tau burden-related change in clustering coefficient is plotted for each group at each node. Red spheres represent local increases as a result of greater overall tau burden; blue spheres represent local decreases. The radius of each sphere is linearly related to the magnitude of disease-related change at that node across the whole range of disease burden observed in each group. All groups are scaled identically. Control images are based on the whole-brain method of assessing tau burden. (**H**) Average raw values for clustering coefficient within each group. The control mean for each metric is subtracted at every node. Red spheres represent increases compared to the control mean value. Blue spheres represent decreases. The centre of the sphere is placed at the centre of the region of interest. The radius of each sphere is linearly related to the difference from the control mean at that node. All groups are scaled identically.

Similarly, we hypothesized that tau accumulation due to a lack of trophic support would manifest as a negative relationship between ^18^F-AV-1451 and clustering coefficient. In Alzheimer’s disease, the opposite relationship was observed; strongly clustered nodes were more likely to display elevated ^18^F-AV-1451 binding (Pearson’s r = 0.33) (Fig. 5A).

Again, by contrast, in PSP those nodes that displayed elevated ^18^F-AV-1451 were those that had the lowest clustering coefficient (Fig. 5B).

### Alzheimer’s disease and PSP have opposite effects on the strength of cortical functional connectivity

We assessed the impact of the presence of tau on connection strength at a variety of spatial scales. First, we averaged weighted degree across the whole brain, resulting in a single measure for each individual. As the overall number of connections in each individual’s graph was thresholded at an identical network density, this measure represented the average strength of the strongest *x*% of connections. To assess global disease burden in Alzheimer’s disease we averaged ^18^F-AV-1451 BP_ND_ across the whole brain. In PSP, we averaged ^18^F-AV-1451 BP_ND_ across midbrain and basal ganglia, reflecting the more focal distribution of disease and our hypotheses about dissociated functional effects of cortical and subcortical tau (Passamonti *et al.*, 2017). Confirmatory tests demonstrated that the pattern of the moderation analyses below were unchanged if whole-brain average ^18^F-AV-1451 BP_ND_ was used to assess tau burden in PSP. Both methods of assessing tau burden were independently assessed in the control group, with no significant effects demonstrated in either case.

In Alzheimer’s disease, we found a negative correlation between average connection strength and tau burden (Pearson’s r = −0.58, *P* = 0.015) (Fig. 2D). In PSP this relationship was reversed, with average connection strength increasing in line with tau burden (r = 0.65, *P* = 0.004). No relationship was observed in controls (r = −0.10, *P* = 0.75). Moderation analysis confirmed a dissociated relationship between tau and connection strength in Alzheimer’s disease and PSP at all network densities [Δr^2^ = 0.29, *F*(1,30) = 19.0, *P* = 0.0001; after partialling out age (Supplementary Fig. 5A) Δr^2^ = 0.28, *F*(1,30) = 17.9, *P* = 0.0002]. Therefore, while in Alzheimer’s disease the presence of tau pathology causes the strongest functional connections to weaken, in PSP, the presence of tau had the opposite effect, i.e. to strengthen functional connections.

Second, we assessed the distribution of change by repeating the correlation of disease burden against weighted degree at every individual node. We hypothesized that this effect would be greatest in those regions that display the strongest functional connectivity in the healthy brain, and which we have demonstrated to accrue most tau in Alzheimer’s disease. The gradient of this nodewise relationship reflects a measure of local change in weighted degree with disease burden (Fig. 2E). First, we examined whether the whole-brain average relationship could be replicated in these individual gradients, by performing sign tests. For Alzheimer’s disease, a negative relationship was confirmed (Z = −13.0, *P* < 0.0001); for PSP there was a positive relationship (Z = 14.8, *P* < 0.0001); while for controls no relationship was demonstrated using either whole brain (Z = −1.1, *P* = 0.27) or deep brain (Z = 1.1, *P* = 0.27) tau burden.

Next, we assessed whether the functional connectivity change at each node related to local tau burden, by correlating the gradient of the disease-related change in weighted degree with the disease-associated increase in ^18^F-AV-1451 binding potential at each node (i.e. correlating each column of Fig. 2F with the corresponding column of Fig. 2G). A negative correlation between these measures was demonstrated in Alzheimer’s disease (Pearson’s r = −0.30, *P* < 0.0001, Spearman’s rho = −0.24, *P* < 0.0001). This relationship was absent in PSP (Pearson’s r = −0.07, *P* = 0.11, Spearman’s rho = −0.00, *P* = 0.98) and in controls (Pearson’s r = −0.01, *P* = 0.75, Spearman’s rho = −0.01, *P* = 0.80). As well as being present at all examined network densities, the negative correlation between disease-related change in functional connectivity strength and tau burden in Alzheimer’s disease was replicated in nodal connectivity strength, the equivalent unthresholded measure (r = −0.34, *P* < 0.0001, Supplementary Fig. 4B).

Finally, we assessed whether the functional connectivity change at each node related to the strength of its connections in the healthy control brain (i.e. correlating each patient panel of Fig. 2G with the control panel in Fig. 2H). As would be expected from the propensity of highly connected nodes to accrue tau, a negative relationship was demonstrated in Alzheimer’s disease (Pearson’s r = −0.23, *P* < 0.0001, Spearman’s rho = −0.24, *P* < 0.0001). Importantly, however, this relationship explained less variance than AV binding, with which we have demonstrated it to be correlated. In PSP, a positive relationship was demonstrated (Pearson’s r = 0.27, *P* < 0.0001, Spearman’s rho = 0.26, *P* < 0.0001).

In summary, nodes that are constitutionally more strongly connected to a larger volume of cortex are more likely to accrue tau pathology in Alzheimer’s disease but not PSP. This relationship is independent of the connectivity network to which a node belongs. Once present, the tau pathology appears to cause local functional connectivity strength to fall. By contrast, in PSP tau selectively accumulates in midbrain and deep nuclei, which has the consequence of increasing the strength of cortico-cortical functional connectivity, especially in those nodes that are constitutionally highly connected.

### Reorganization of cortical functional connectivity reflects cortical versus subcortical pathology

We hypothesized that the presence of subcortical pathology in PSP might be causing an increase in weighted degree by necessitating an increase in the relative strength of short-range cortico-cortical connections as longer range connections are disrupted. This view is supported by the observation that a consequence of increasing tau burden in PSP is a marked reduction in participation coefficient (Fig. 4D). This relationship was not observed in Alzheimer’s disease, where increasing tau burden caused increasing participation. Moderation analysis confirmed this differential relationship at all network densities from 1% to 10% [Δr^2^ = 0.21, *F*(1,30) = 7.6, *P* = 0.002; after partialling out age (Supplementary Fig. 5B) Δr^2^ = 0.17, *F*(1,30) = 8.3, *P* = 0.007].

We further examined this hypothesis by examining the effect of tau on other measures of graph structure in each individual, dissociated from variation in degree by binarization after thresholding and normalization against 1000 random graphs with the same number of connections at each node.

The clustering coefficient quantifies how many of a node’s neighbours are neighbours of each other. All individuals in all groups displayed clustering to at least 2.8× that of random graphs of the same degree (Fig. 5D), consistent with a small-world connectivity distribution. While in Alzheimer’s disease a non-significant trend was observed towards reduced clustering with increasing tau burden, in line with previous reports (Stam *et al.*, 2006; Sanz-Arigita *et al.*, 2010), in PSP the opposite relationship were observed. Moderation analysis trended towards significance at the density of primary interest [Δr^2^ = 0.08, *F*(1,30) = 3.8, *P* = 0.06; after partialling out age (Supplementary Fig. 5C) Δr^2^ = 0.07, *F*(1,30) = 2.5, *P* = 0.09], and was significant from 7% to 10% density. The betweenness centrality of a node is a measure of the number of shortest paths between any other two nodes that pass through it. If the presence of subcortical pathology in PSP means that long-range information transfer must occur through a transcortical route, average betweenness centrality should increase. Conversely, predominantly cortical pathology in Alzheimer’s disease might increase reliance on cortico-subcortical connections, reducing average betweenness centrality. This prediction was verified (Supplementary Fig. 6A), with a differential relationship between tau burden and betweenness centrality confirmed by moderation analysis at all network densities from 1–10% [Δr^2^ = 0.21, *F*(1,30) = 9.3, *P* = 0.005; after partialling out age (Supplementary Fig. 5D) Δr^2^ = 0.20, *F*(1,30) = 8.9, *P* = 0.006].

**Figure 6:**
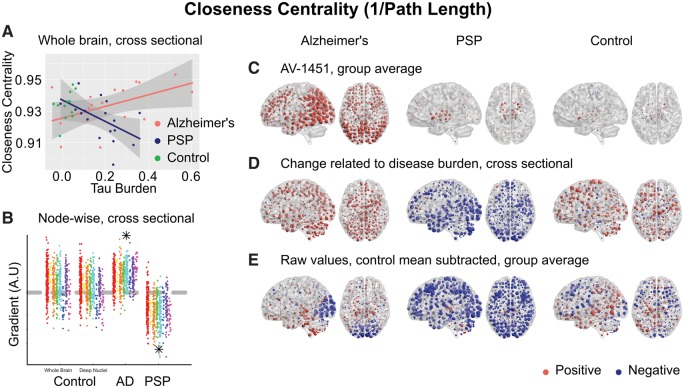
**Closeness centrality (1/path length from each node to all other nodes).** (**A**) Between-subjects analysis of the relationship between global tau burden and closeness centrality at a network density of 6%. No significant relationship was found for the control subjects with either method of assessing tau burden. Moderation analysis for a differential relationship between graph metric and tau burden in the two disease groups [Alzheimer’s disease (AD) and PSP] was statistically significant. (**B**) The magnitude of disease related change at each node is plotted as a single point, grouped by lobe. Control data are shown for both methods of assessing tau burden. Stars represent statistically significant excesses of positive or negative gradients in the disease groups (Supplementary Table 2). (**C**) Average ^18^F-AV-1451 binding potential at each node. Red spheres represent increases compared to the cerebellar reference region. Blue spheres represent decreases. The centre of the sphere is placed at the centre of the region of interest. The radius of each sphere is linearly related to the magnitude of binding at that node. All groups are scaled identically. (**D**) The local tau burden-related change in closeness centrality is plotted for each group at each node. Red spheres represent local increases as a result of greater overall tau burden; blue spheres represent local decreases. The radius of each sphere is linearly related to the magnitude of disease-related change at that node across the whole range of disease burden observed in each group. All groups are scaled identically. Control images are based on the whole-brain method of assessing tau burden. (**E**) Average raw values for closeness centrality within each group. The control mean for each metric is subtracted at every node. Red spheres represent increases compared to the control mean value. Blue spheres represent decreases. The centre of the sphere is placed at the centre of the region of interest. The radius of each sphere is linearly related to the difference from the control mean at that node. All groups are scaled identically.

Long-range information transfer by a cortico-cortical route implies an inefficient, indirect process, perhaps accounting for the cognitive slowing characteristic of neurodegenerative disorders with predominant subcortical pathology. We tested for this by examining closeness centrality, which is the inverse of the path length between a node and all other nodes in the graph. As would be predicted by this account, increasing disease burden resulted in a higher average path length (lower closeness centrality) in PSP but a lower average path length in Alzheimer’s disease (higher closeness centrality) at network densities from 2% to 10% [Fig. 6A, moderation Δr^2^ = 0.20, *F*(1,30) = 9.1, *P* = 0.005; after partialling out age (Supplementary Fig. 5E) Δr^2^ = 0.16, *F*(1,30) = 8.0, *P* = 0.008]. Averaged across the whole brain, this measure is equivalent to the global efficiency of the graph, and indeed the same statistical results were obtained from a moderation analysis of that metric; increasing disease burden resulted in reduced global efficiency in PSP.

**Figure 7:**
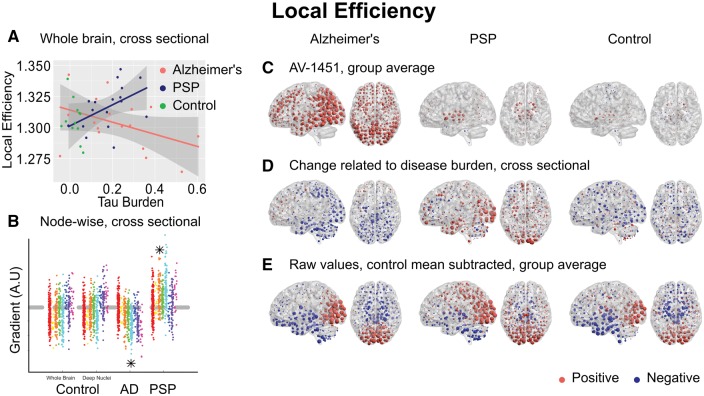
**Local efficiency.** (**A**) Between-subjects analysis of the relationship between global tau burden and local efficiency at a network density of 6%. No significant relationship was found for the control subjects with either method of assessing tau burden. Moderation analysis for a differential relationship between graph metric and tau burden in the two disease groups [Alzheimer’s disease (AD) and PSP] was statistically significant. (**B**) The magnitude of disease related change at each node is plotted as a single point, grouped by lobe. Control data are shown for both methods of assessing tau burden. Stars represent statistically significant excesses of positive or negative gradients in the disease groups (Supplementary Table 2). (**C**) Average ^18^F-AV-1451 binding potential at each node. Red spheres represent increases compared to the cerebellar reference region. Blue spheres represent decreases. The centre of the sphere is placed at the centre of the region of interest. The radius of each sphere is linearly related to the magnitude of binding at that node. All groups are scaled identically. (**D**) The local tau burden-related change in local efficiency is plotted for each group at each node. Red spheres represent local increases as a result of greater overall tau burden; blue spheres represent local decreases. The radius of each sphere is linearly related to the magnitude of disease-related change at that node across the whole range of disease burden observed in each group. All groups are scaled identically. Control images are based on the whole-brain method of assessing tau burden. (**E**) Average raw values for local efficiency within each group. The control mean for each metric is subtracted at every node. Red spheres represent increases compared to the control mean value. Blue spheres represent decreases. The centre of the sphere is placed at the centre of the region of interest. The radius of each sphere is linearly related to the difference from the control mean at that node. All groups are scaled identically.

While tau-mediated reorganization of connectivity in PSP results in slower and less efficient long-range information transfer, in Alzheimer’s disease one might suppose it to be a beneficial consequence of the loss of overall connection strength as those connections that remain are more globally efficient. However, we hypothesized this would be at the cost of local efficiency, which is a measure of the number of strong connections between neighbouring nodes and the robustness of local networks to disruption. Indeed this was found to be the case at network densities from 4% to 10% [Fig. 7A, Δr^2^ = 0.15, *F*(1,30) = 6.5, moderation *P* = 0.016; after partialling out age (Supplementary Fig. 5F) Δr^2^ = 0.12, *F*(1,30) = 5.5, *P* = 0.026].

Finally, we assessed the functional influence of each node on the other nodes in the network by examining the eigenvector centrality. Again, an opposite effect of disease burden was observed in Alzheimer’s disease and PSP at network densities from 3–10% [Supplementary Fig. 7A, Δr^2^ = 0.22, *F*(1,30) = 8.5, *P* = 0.007; after partialling out age (Supplementary Fig. 5G) Δr^2^ = 0.20, *F*(1,30) = 8.3, *P* = 0.007] such that, on average, as PSP progressed each node had less functional influence on every other node in the graph.

### Local functional connectivity reorganization is related to tau in Alzheimer’s disease

To understand the differential reorganization of functional connectivity in Alzheimer’s disease and PSP, we replicated the approach taken for weighted degree; correlating the gradient of the disease-related change at every individual node with local change in ^18^F-AV-1451 binding potential. For all metrics, the differential group effects demonstrated by moderation analysis could be replicated by statistical significance of opposite directionality in sign tests performed on the individual gradients at each node (Supplementary Table 2, Figs 4E, 5E, 6B, 7B and Supplementary Figs 6B and 7B). In all cases, controls showed either no relationship or a weaker effect in the same direction as patients with Alzheimer’s disease.

The reorganization of graph metrics followed two distinct patterns. Closeness centrality (Fig. 6D) displayed a global effect, with most nodes increasing in Alzheimer’s disease and decreasing in PSP. This was not strongly related to local ^18^F-AV-1451 binding potential in Alzheimer’s disease (Pearson’s r = 0.05, *P* = 0.25, Spearman’s rho = 0.06, *P* = 0.18) or PSP (Pearson’s r = 0.08, *P* = 0.06, Spearman’s rho = 0.11, *P* = 0.01). Similarly, participation coefficient displayed global changes in both diseases (Fig. 4G) (Alzheimer’s disease Pearson’s r = 0.09, Spearman’s rho = 0.07, PSP Pearson’s r = 0.08, Spearman’s rho = 0.06).

By contrast, eigenvector centrality (Supplementary Fig. 7D) was more strongly related to local ^18^F-AV-1451 binding potential in Alzheimer’s disease (Pearson’s r = −0.28, *P* < 0.0001, Spearman’s rho = −0.25, *P* < 0.0001). Strikingly, the positive relationship we demonstrated across the whole brain masked opposing regional effects. As global tau burden increased, the functional influence of frontal regions on all other regions increased, while that of occipital regions decreased. In PSP, no consistent relationship with ^18^F-AV-1451 was observed (Pearson’s r = −0.10, *P* = 0.02, Spearman’s rho = −0.04, *P* = 0.38), with almost all regions having less functional influence on all other regions as tau burden increased.

Clustering coefficient (Fig. 5G), local efficiency (Fig. 7D) and betweenness centrality (Supplementary Fig. 6D) displayed an intermediate degree of regional specificity, being weakly but significantly correlated with change in ^18^F-AV-1451 binding potential in Alzheimer’s disease (clustering coefficient Pearson’s r = −0.12, *P* < 0.0001, Spearman’s rho = −0.14, *P* < 0.0001, local efficiency Pearson’s r = −0.16, *P* < 0.0001, Spearman’s rho = −0.19, *P* < 0.0001, betweenness centrality Pearson’s r = 0.19, *P* < 0.0001, Spearman’s rho = 0.19, *P* < 0.0001) and PSP (clustering coefficient Pearson’s r = −0.14, *P* < 0.0001, Spearman’s rho = −0.12, *P* < 0.0001, local efficiency Pearson’s r = −0.14, *P* = 0.001, Spearman’s rho = −0.11, *P* = 0.02, betweenness centrality Pearson’s r = 0.17, *P* < 0.0001, Spearman’s rho = 0.08, *P* = 0.08).

## Discussion

We have demonstrated that in Alzheimer’s disease a strong relationship exists between the propensity of a node to display elevated ^18^F-AV-1451 binding and the volume of cortex to which it is strongly connected (Fig. 2A). Further, we have demonstrated that this effect exists both within and between intrinsic connectivity networks (Fig. 3). This is consistent with the theory of trans-neuronal spread. The predictions of the competing hypotheses that highly active brain regions are vulnerable to tau accumulation due to a positive relationship with metabolic demand (Fig. 4A) or negative relationship with clustering due to a lack of trophic support (Fig. 5A) were not supported by the data.

In contrast, in PSP, we have demonstrated the opposite findings. Those brain regions that accrue most tau display weak connectivity (Fig. 2B), but are predicted to have high metabolic demand (Fig. 4B) and a lack of trophic support (Fig. 5B).

Further, we have explored the consequences of tau accumulation with cross-sectional analyses at a variety of spatial scales. In Alzheimer’s disease, we have demonstrated that with greater levels of tau pathology the strongest internodal connections are weakened (Fig. 2G). This reorganization of the brain network leads to more direct long-range connections passing through fewer nodes (Fig. 6A and Supplementary Fig. 6A), at the cost of lower local efficiency (Fig. 7A)

In PSP, where tau accumulation is predominantly subcortical, we demonstrate the opposite reorganization of the connectivity graph. With greater levels of tau in midbrain and deep nuclei (Fig. 2F), the strongest functional connections are strengthened; these are predominantly cortical (Fig. 2G and H). Information transfer therefore takes a less direct path (Supplementary Fig. 6A), passing through a larger number of cortical nodes *en route* (Fig. 4A), as deep structures can no longer sustain long range connectivity. This is accompanied by a decrease in participation coefficient (Fig. 4D) and an increase in clustering (Fig. 5D) as connectivity becomes increasingly modular.

### Insights into the mechanisms of disease progression in humans

It has been proposed that the pathological mechanisms underlying Alzheimer’s disease begin in a single, vulnerable location and spread from cell to cell, rather than occurring independently in a large number of vulnerable cell populations (Guo and Lee, 2014; Goedert, 2015). The primary direct evidence for such propagation of tau comes from rodent studies. For example, the injection of brain extract from transgenic mice expressing mutant tau into mice expressing wild-type human tau caused wild-type tau to form filaments and spread to neighbouring brain regions (Clavaguera *et al.*, 2009). Further, pathological tau from human brains causes disease in wild-type mice, in which the pathological human tau species becomes self-propagating (Clavaguera *et al.*, 2013). This tau propagation is mediated by the presence and strength of synaptic connectivity rather than spatial proximity (Liu *et al.*, 2012; Iba *et al.*, 2013; Ahmed *et al.*, 2014).

Associative studies of the healthy brain have demonstrated that large-scale, functionally connected neural networks strongly resemble the known patterns of atrophy in distinct neurodegenerative syndromes mediated by tau and TDP-43 (Zhou *et al.*, 2012). Further human evidence comes from the observation that patterns of atrophy in the rare disease non-fluent variant primary progressive aphasia (nfvPPA) strongly correspond to structural and functional connectivity in the healthy speech production network (Mandelli *et al.*, 2016). This is an important observation, because clinically and radiologically indistinguishable cases of nfvPPA can be caused either by tau or by the unrelated protein TAR DNA-binding protein 43 (TDP-43), which has been demonstrated to propagate trans-neuronally (Braak *et al.*, 2013).

Here, we go beyond these associative studies to measure tau burden and functional connectivity in the same individuals at both the whole-brain and regional level. Our observation that those brain areas that are more strongly functionally connected have accrued more tau pathology in Alzheimer’s disease (Fig. 2A), independent of which connectivity network they belong to (Fig. 3), is consistent with trans-neuronal spread. We demonstrate that the presence of tau is not, in itself, inducing stronger regional connectivity by our cross-sectional analysis of the Alzheimer’s disease group, in which we demonstrate that as cortical tau accumulates the overall functional connectivity of cortex falls (Fig. 2D), and this between-subjects effect is strongest in those brain regions with most tau accumulation (Fig. 2F and G). Crucially, we demonstrate that ^18^F-AV-1451 binding potential at each node is better than the connectivity of that node in the healthy brain at accounting for regional variance in connectivity change, arguing against the presence of tau being a secondary marker of neurodegeneration in vulnerable hubs. In other words, it is not coincidence that Alzheimer’s disease tends to impact large networks; it is a predictable consequence of trans-neuronal spread of a disease-causing protein. Graph theoretic models of transmissible disease epidemics are in agreement that the likelihood of an individual becoming infected (and the dose of the infectious agent received) is directly proportional to its number of infected neighbours and their infectivity (Durrett, 2010). As our nodes represent brain regions of equal volume, the binary portion of degree represents a surrogate measure of the number of neurons to which a brain region is connected, and the weighted portion of degree is a measure of the strength of these connections. By the time Alzheimer’s disease is sufficiently advanced to cause the symptoms of MCI, tau is generally already present to some degree throughout the neocortex (Markesbery, 2010), and therefore reaching a disease stage at which the number of neighbours more closely approximates the number of infected neighbours, and the connection strength between infected neighbours (here the weighted portion of degree) becomes a strong driver of infectivity.

Conversely, our analyses do not provide support for models of hub vulnerability due to metabolic demand or lack of trophic support in Alzheimer’s disease. It is important to acknowledge that this does not mean that these mechanisms are unimportant, but rather that they are a downstream event of tau accumulation. In other words, while we demonstrate that the propensity of a node to accrue tau is not related to metabolic demand or trophic support, these factors might still contribute to determining the vulnerability of brain regions to the presence of a given amount of tau. This hypothesis could be addressed in future studies by relating the information content of tau ligand binding to other measures of neurodegeneration such as longitudinal changes in grey matter volume.

In PSP, we do find support for models of hub vulnerability due to metabolic demand and lack of trophic support, but not for models of trans-neuronal spread. This is in line with other recent studies, demonstrating that neurodegeneration in PSP is related to local gene expression patterns (Rittman *et al.*, 2016).

### Is the difference between Alzheimer’s disease and PSP mediated by tau isoform or intrinsic connectivity?

A striking feature of our results is that we demonstrate a strong relationship between ^18^F-AV-1451 binding potential and the strength of functional connectivity in Alzheimer’s disease but not PSP. There are at least two potential (and not mutually exclusive) explanations for this dissociation.

The first possibility is that trans-neuronal tau propagation might occur more slowly in PSP. Cellular models have demonstrated that the propensity of tau to propagate intracellularly depends on its ability to form aggregates (Falcon *et al.*, 2015). Once present in a new cell, the ability of tau fibrils to induce the aggregation of constitutionally present tau depends on the conformation of the fibril structure (Nonaka *et al.*, 2010; Fitzpatrick *et al.*, 2017). It might be that the straight filaments of predominantly 4R tau that characterize PSP are simply less able to propagate or need to be present in higher concentrations before they can induce a chain reaction of local tau aggregation (Guo *et al.*, 2016). This view is supported by our demonstration that tau is restricted to brainstem and deep nuclei in PSP despite these nodes being highly promiscuous between networks, displaying a high participation coefficient (Fig. 4B) and low clustering (Fig. 5B).

Second, it is possible that tau propagation does occur, but that it is limited in range to a subcortical intrinsic connectivity network (Raj *et al.*, 2012) (perhaps network 5 in Laird *et al.*, 2011). Such a subcortical network may be poorly visualized by multi-echo functional MRI, and is less frequently observed at rest than the DMN (Greicius *et al.*, 2003), which has been implicated in Alzheimer’s disease (Greicius *et al.*, 2004). Together, therefore, our findings of weak functional connectivity in deep nuclei and brainstem nodes (Fig. 2B) might mask meaningful functional connectivity within and between these regions, accounting for the restricted and stereotyped pattern of tau accumulation in early PSP (Ellison *et al.*, 2012) and cortical escape in advanced PSP (Schofield *et al.*, 2012).

### Cross-sectional data reveals patterns less visible at the group level

Computational modelling of connectivity-dependent cell death predicts a dissociation between changes in functional connectivity in early and late Alzheimer’s disease (de Haan *et al.*, 2012). It has been proposed that, in early disease, hubs compensate for declines in structural connectivity by increasing their firing rate, manifesting as stronger functional connectivity (Maestú *et al.*, 2015). As disease progresses, this mechanism breaks down as neural damage prevents the maintenance of this metabolically demanding compensation (Jones *et al.*, 2015). This dissociation is thought to underlie some of the seemingly inconsistent findings in the analysis of graph properties in neurodegenerative disease. By using a cross-sectional approach across a range of disease severity, we demonstrate relationships consistent with this hypothesis. In Alzheimer’s disease, weighted degree (Fig. 2D) and betweenness centrality (Supplementary Fig. 6A) consistently fall as tau burden increases. However, in early disease where tau burden is low (our sample includes a range of severity, including PiB-positive MCI), the regression line for these metrics is above the control average. Similarly, examining the regional changes related to disease burden reveals striking patterns that are obscured at the group level. This underlines the more general principal that one should be cautious in interpreting a main effect of group in the presence of an interaction, or correlation with severity.

### Reorganization of brain networks

Our examination of two distinct tau-mediated neurodegenerative pathologies with different distributions of pathology has enabled us to distinguish their consequences. We demonstrate strongly opposing effects in a range of metrics resulting from the presence of predominantly cortical (Alzheimer’s disease) or subcortical (PSP) tau. The direction of these effects in Alzheimer’s disease is consistent with the previously recognized impact of neurodegeneration, leading to increasingly random cortical connectivity (Sanz-Arigita *et al.*, 2010; Stam, 2014) and a reduction in small-world properties (Stam *et al.*, 2006). Eigenvector centrality showed particularly strong regional effects in Alzheimer’s disease, with a negative correlation observed between disease-related changes in this metric and local tau burden at each node—i.e. those brain regions that displayed less tau pathology had greater functional influence on other brain regions.

In contrast, cortico-subcortical functional connectivity is preferentially impaired in PSP-Richardson’s syndrome, resulting in cerebral information transfer taking a less direct path through a larger number of cortical nodes, reducing closeness centrality and eigenvector centrality, but increasing cortical degree, betweenness centrality and local efficiency. This results in an excessively modular connectivity arrangement, with decreasing participation coefficient and increasing clustering. These findings tie together classical observations of the ‘subcortical dementia’ phenotype of PSP (Albert *et al.*, 1974) with more modern observations that behavioural change and cognitive impairment in PSP correlates with frontal cortical hypometabolism (D'Antona *et al.*, 1985; Foster *et al.*, 1988) and atrophy (Cordato *et al.*, 2002, 2005). We propose that increases in cortical functional connectivity can compensate for subcortical tau burden in PSP. Increasingly indirect information transfer accounts for the cognitive slowing that is the hallmark of the ‘subcortical dementias’, but performance on untimed tests is preserved until cortical regions become atrophic in late disease.

### Study limitations

The main limitation of our analysis is that it is cross-sectional, and we use ^18^F-AV-1451 binding as a surrogate marker of tau burden. By making observations about the relationship between tau burden and functional connectivity in this way, we assume a uniformity of effect within our disease groups. As novel PET ligands such as ^18^F-AV-1451 gain maturity, longitudinal assessment of tau burden and functional connectivity in the same individuals will be an important and powerful validation of our results. Definite evidence of the causal relationship between tau and connectivity with require the combination of longitudinal assessment and interventional studies targeting tau pathology. It should also be noted that ^18^F-AV-1451 binding identifies predominantly aggregated tau in tangles, and does not directly measure oligomeric tau, which may be more toxic to the cell and synaptic plasticity, nor extracellular forms of tau that may mediate spread of pathology. The molecular binding target of ^18^F-AV-1451 in non-Alzheimer’s disease tauopathies is disputed; elevated ‘off-target’ binding has been demonstrated in the basal ganglia of healthy controls (Johnson *et al.*, 2016), albeit to a lesser degree than that observed in PSP (Fig. 2F) (Passamonti *et al.*, 2017), and in TDP-43 associated disorders without evident tau pathology (Bevan-Jones *et al.*, 2017*a*). Nonetheless, ^18^F-AV-1451 is able to recapitulate the distribution of post-mortem neuropathology in these disorders, making it appropriate for use here (Smith *et al.*, 2016).

Our analysis is focused towards cortico-cortical functional connectivity. In particular, multi-echo MRI might have a poor signal-to-noise ratio in deep brain structures, although the main advantage of using this sequence is that it enables robust de-noising of movement-related artefacts pipeline (Kundu *et al.*, 2012, 2013). This is critical in clinical populations, in which functional MRI data may differentially suffer from quality degradation due to head movements.

Finally, by examining proportionately thresholded graphs with 1–10% density, our analysis focusses on the strongest interregional functional connections. However, it is possible that we are missing additional effects of neurodegeneration on weak or medium-strength connections. Tract-tracing studies indicate that there are weak anatomical connections, equivalent to a few axons, between some cortical areas (Ypma and Bullmore, 2016). Such weak links may have functional importance in complex networks (Granovetter, 1983). However, weak connections are difficult to evaluate with functional MRI, as it is not possible to disentangle them from correlation arising from signal noise. Future evaluation of these weaker connections with *in vivo* tractography, neuropathology or novel methods might reveal additional effects not evident in our dataset. In the interim, the thresholding procedure retains several advantages; by retaining only the most strongly correlated edges one is less likely to include false positive correlations and topologically random edges. It also allows the computationally intense process of normalization of metrics against random graphs of equal density. The consistency between the results using thresholded nodal weighted degree and unthresholded nodal connectivity strength provides reassurance in the choice of thresholding of connections.

## Conclusion

This study reveals the differential relationship between tau burden and functional connectivity in two distinct human neurodegenerative tauopathies. Our results enable us to disentangle the causes of tau accumulation from their consequences. They have wide-ranging implications, from the validation of models of tau trafficking in humans to corroborating computational models of hub compensation in Alzheimer’s disease, while accounting for the contrasting cognitive phenotype of these two conditions. These insights into the relationship between tau burden and brain connectivity changes will inform translational models and clinical trials of disease-modifying therapies.

## Supplementary Material

Supplementary Figure S1

Supplementary Figure S2

Supplementary Figure S3

Supplementary Figure S4

Supplementary Figure S5

Supplementary Figure S6

Supplementary Figure S7

Supplementary Figure Legends

## Abbreviations

BP_ND_: binding potential of PET ligand
MCI: mild cognitive impairment
PSP: progressive supranuclear palsy
