# Supplementary figures and images for: Tau burden and the functional connectome in Alzheimer’s disease and progressive supranuclear palsy
Source: Brain. 2018 Jan 5;141(2):550–67. doi: 10.1093/brain/awx347 (PMC5837359; doi:10.1093/brain/awx347)

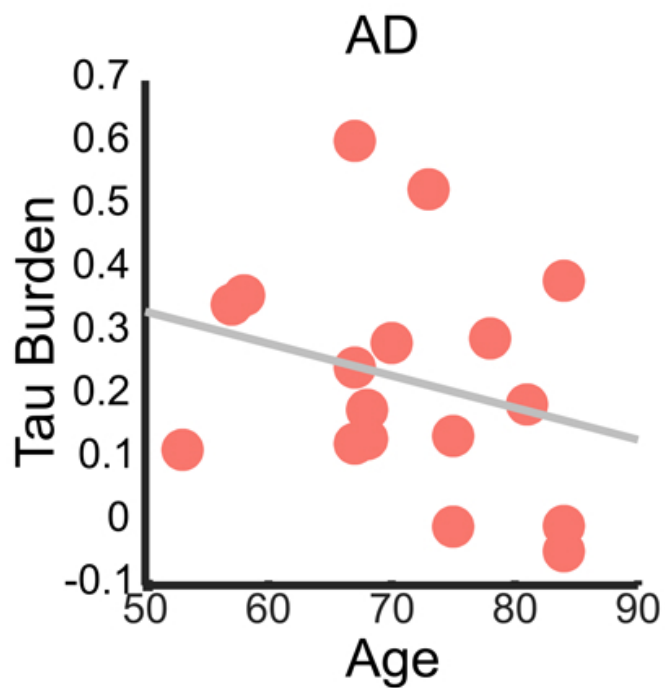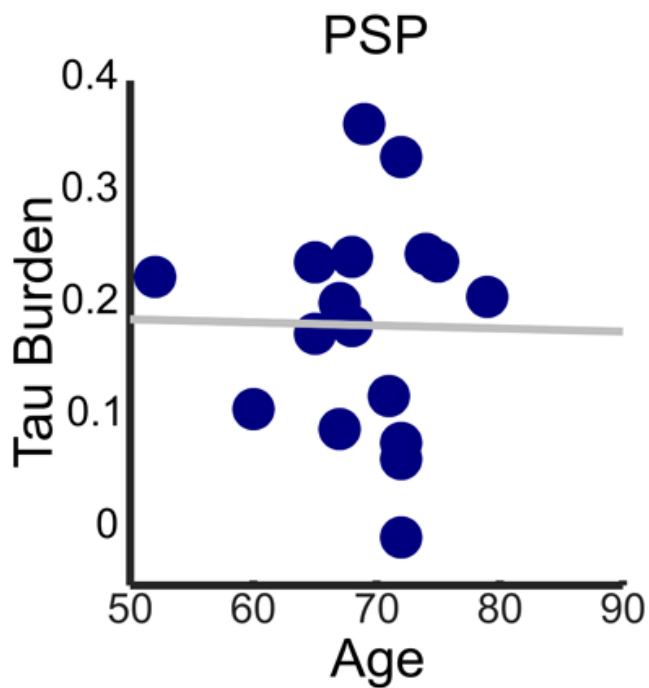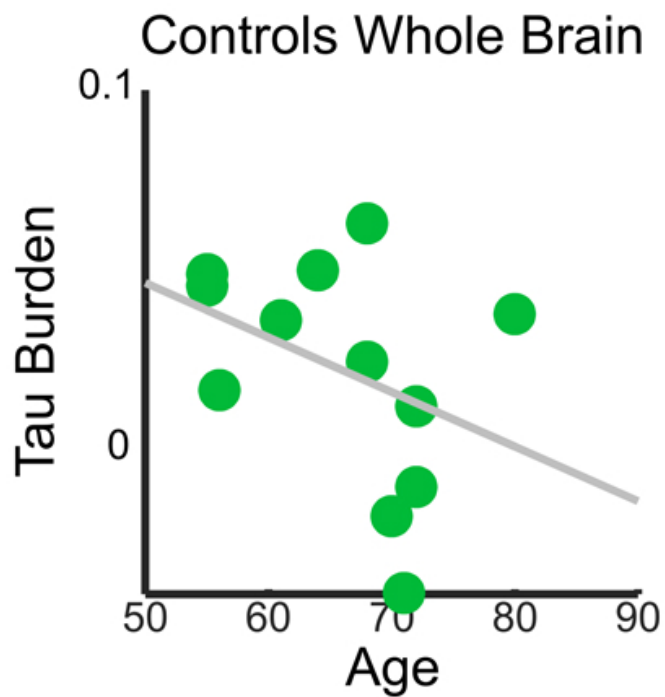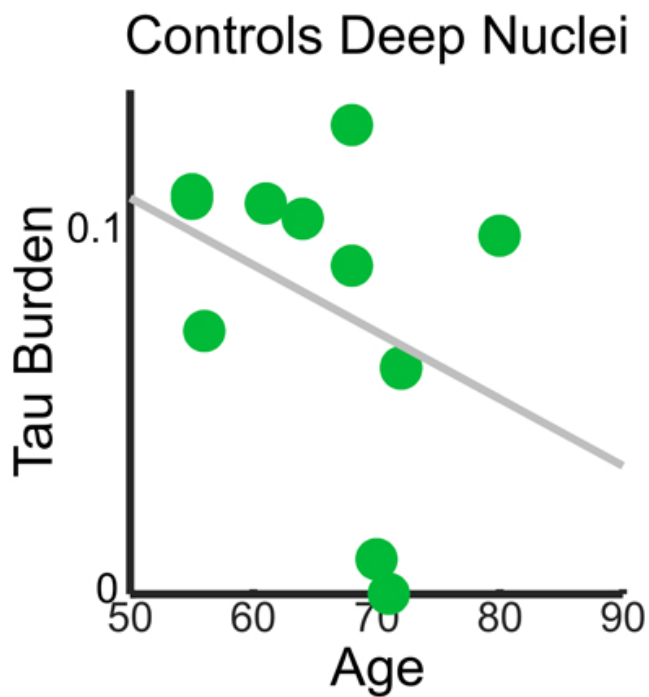

Supplement: Supplementary Figure S2 [file brain-2017-01363-file010_awx347.pdf]

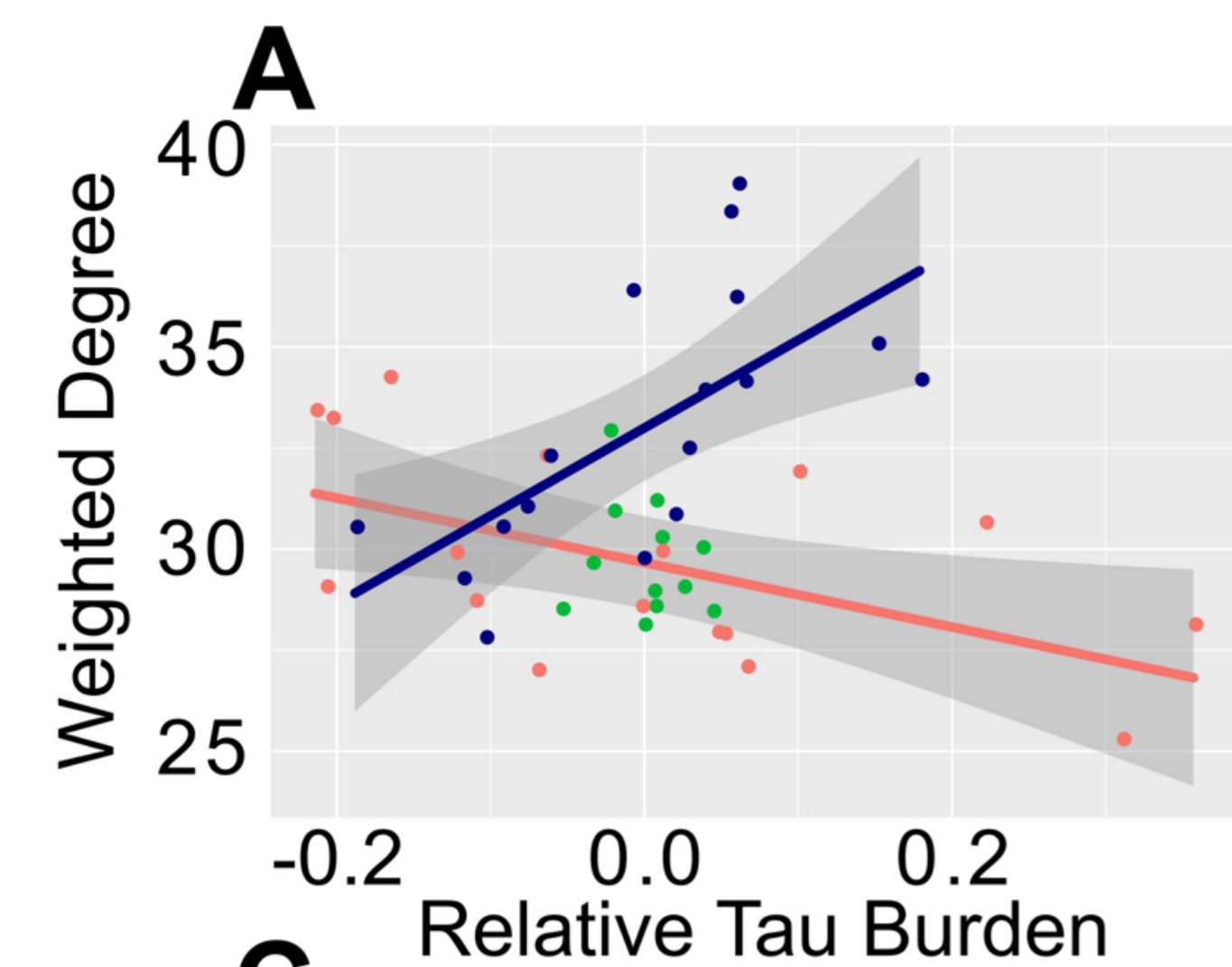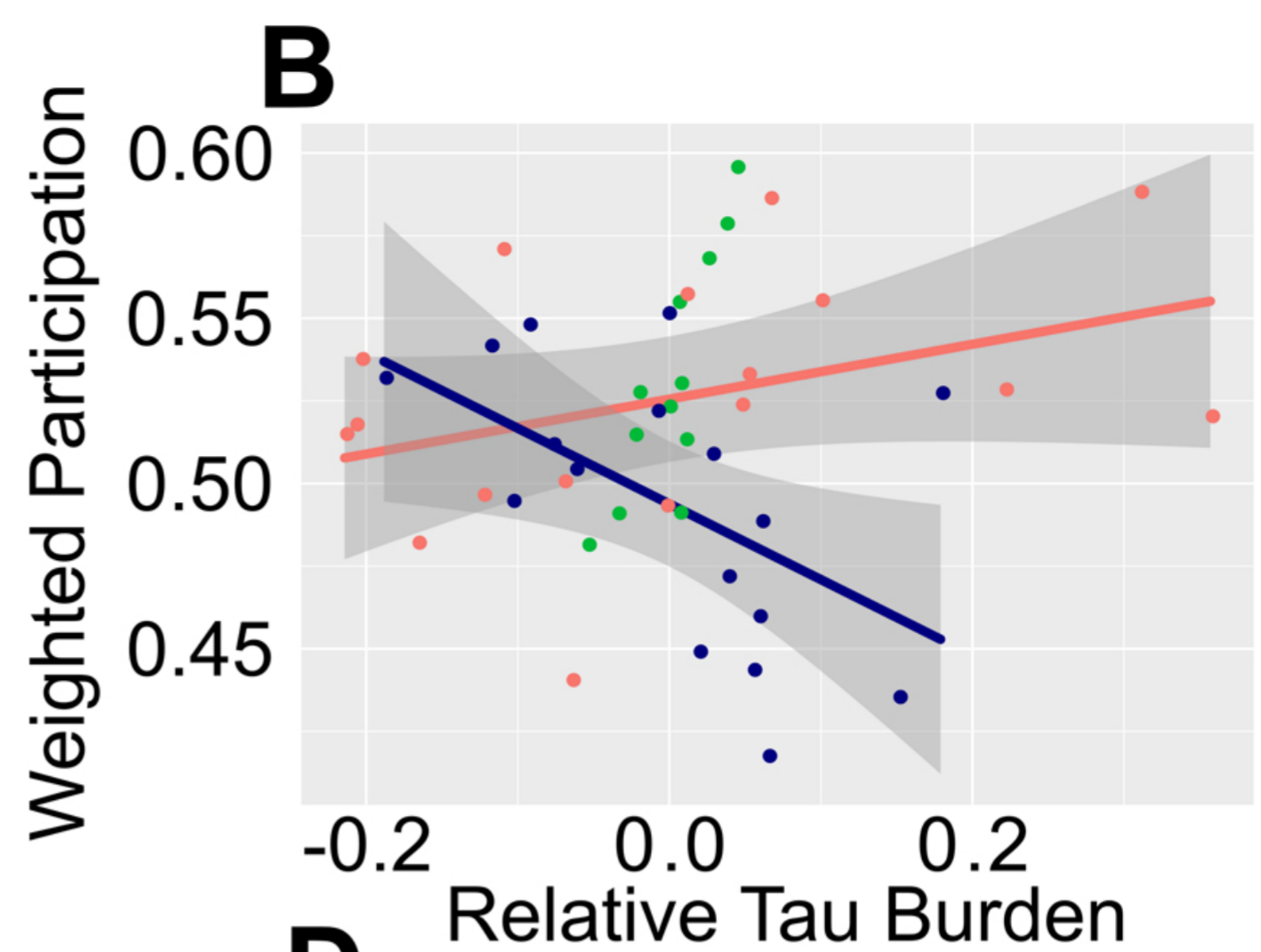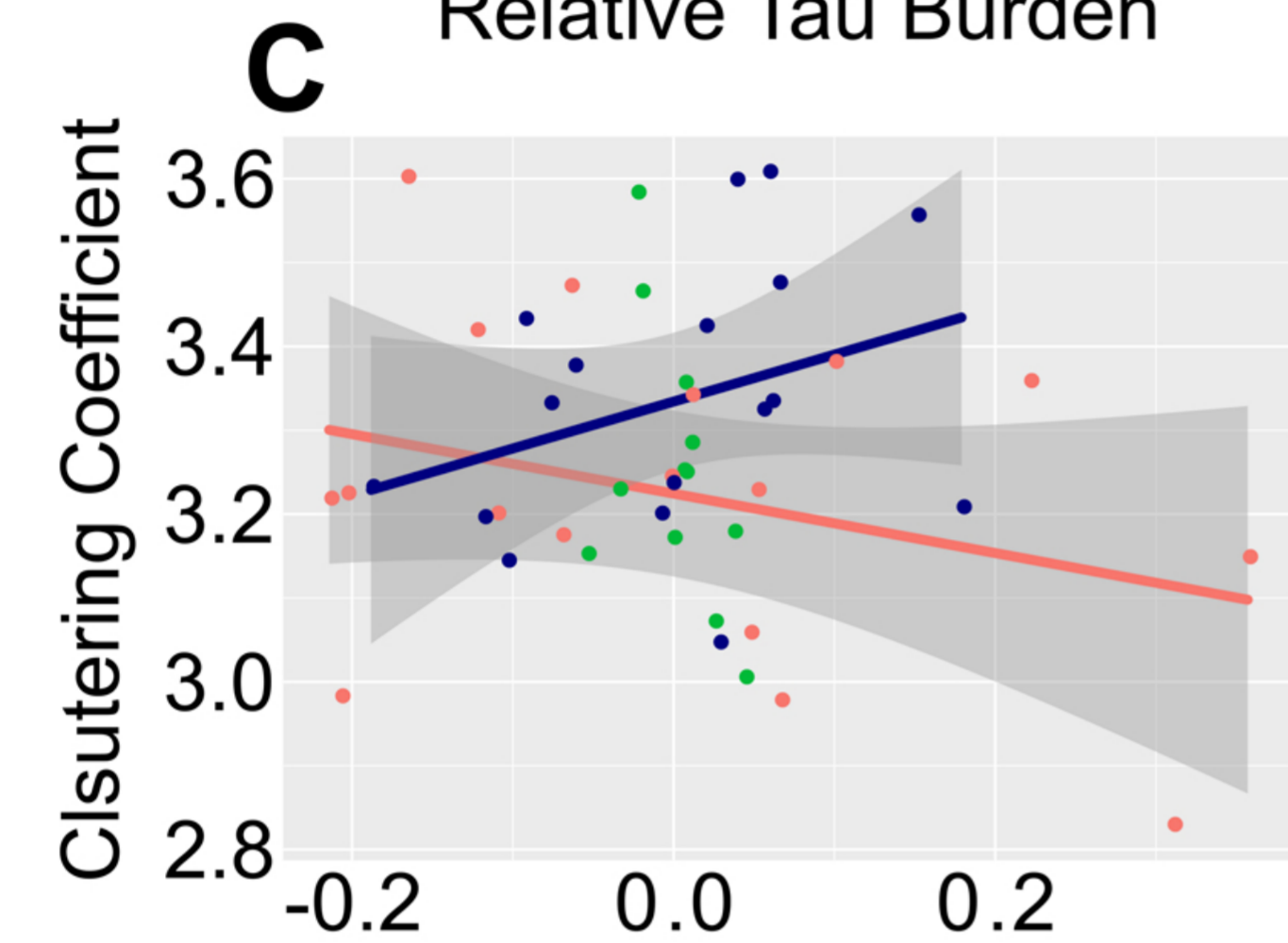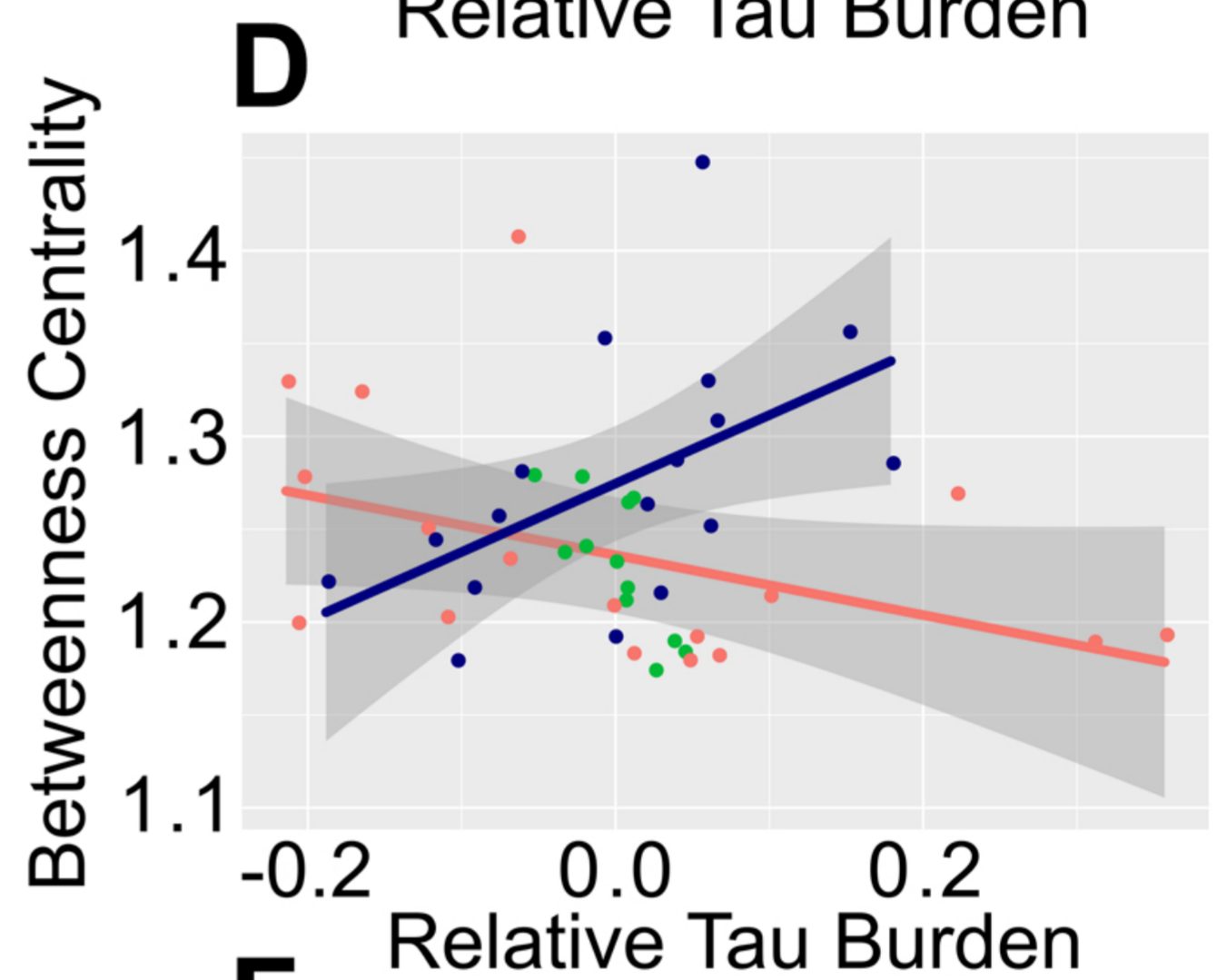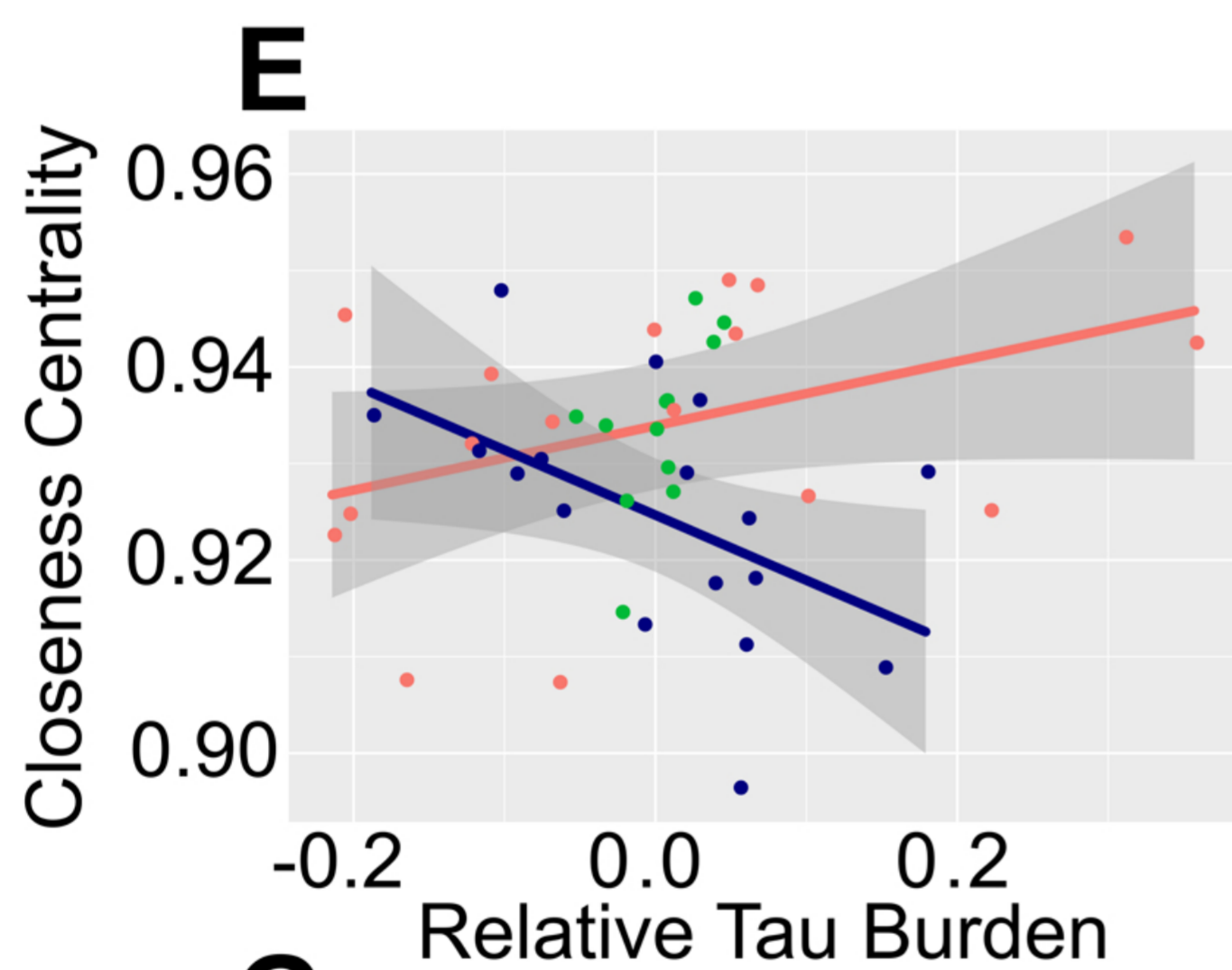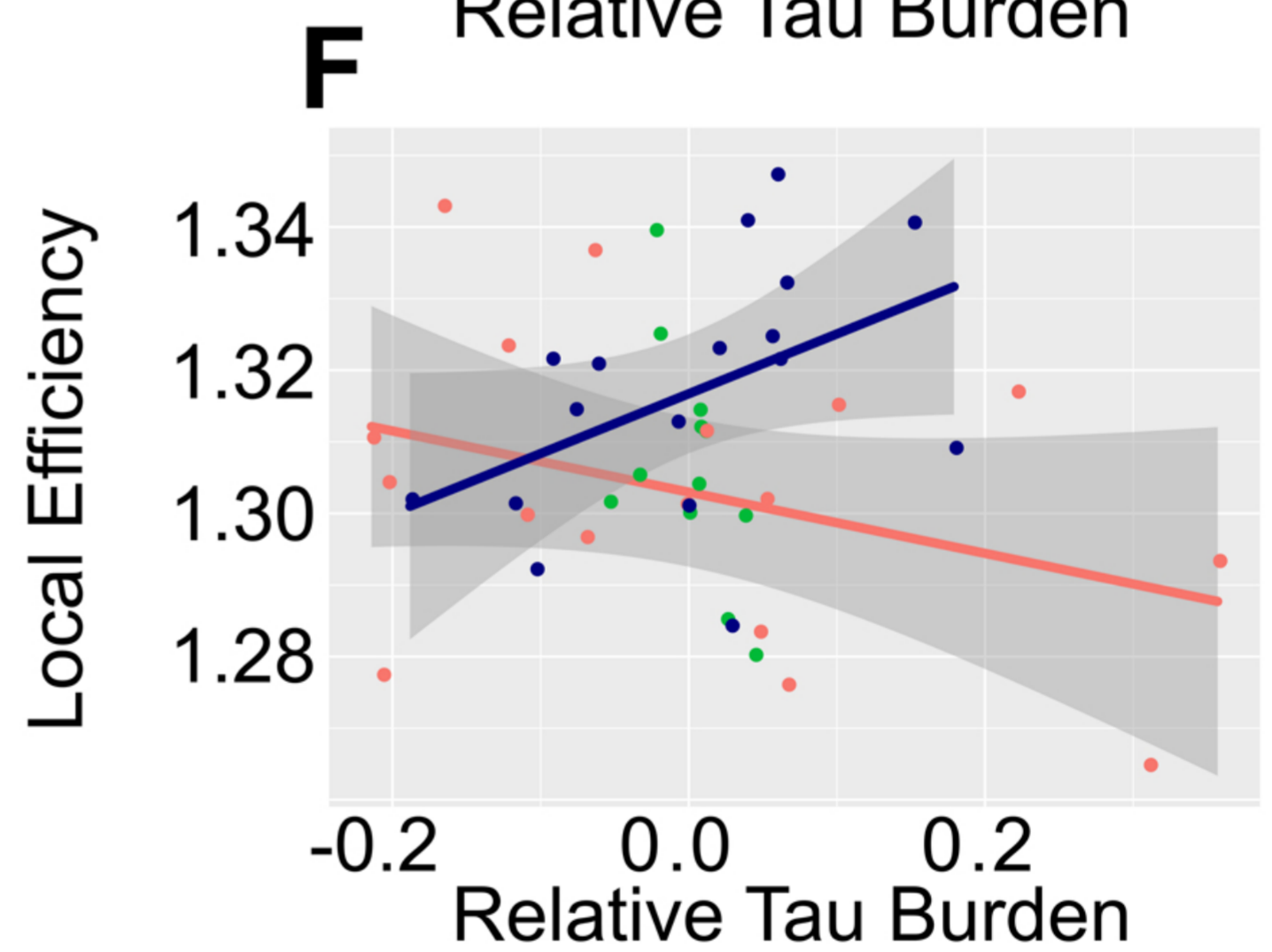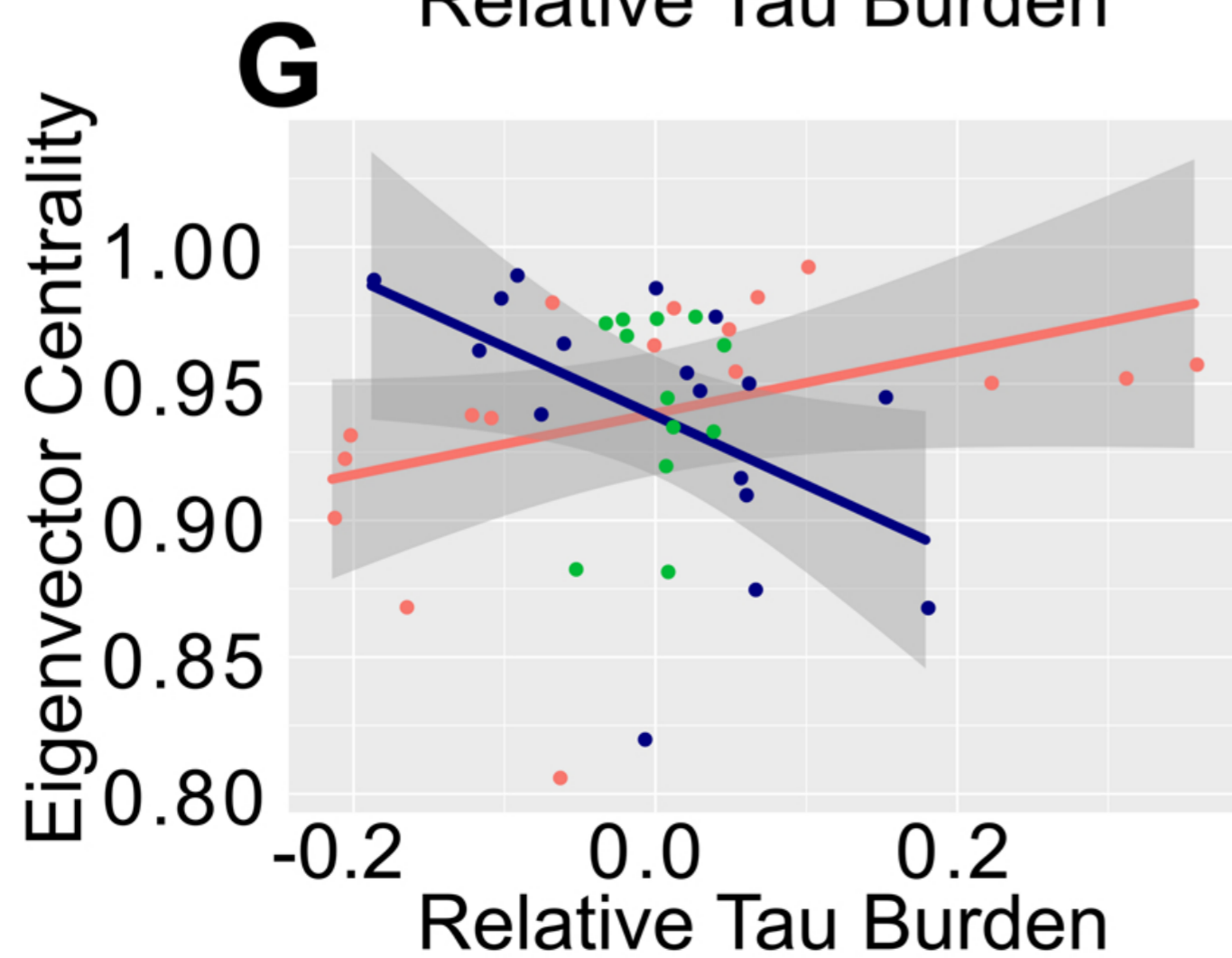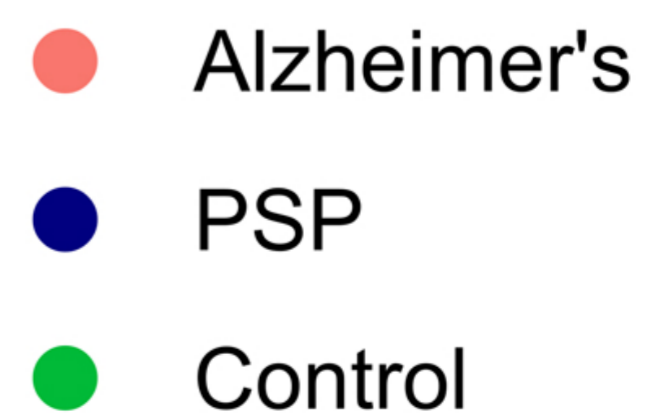

Supplement: Supplementary Figure S3 [file brain-2017-01363-file011_awx347.pdf]

# PSP

- Frontal
- Insula
- Temporal
- Parietal
- Occipital
- Deep nuclei
- Cerebellum
- Brainstem

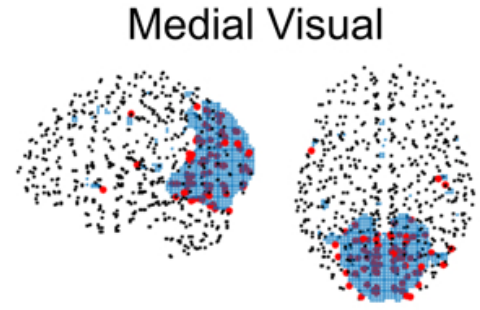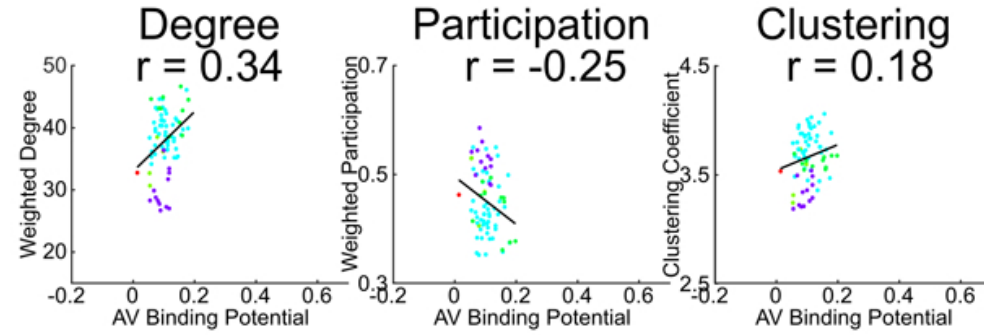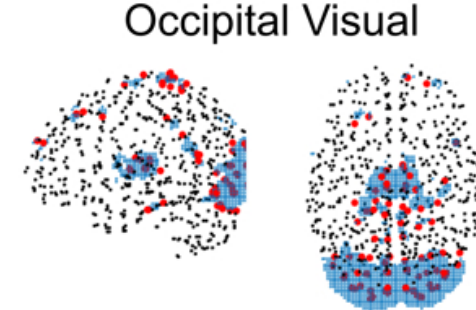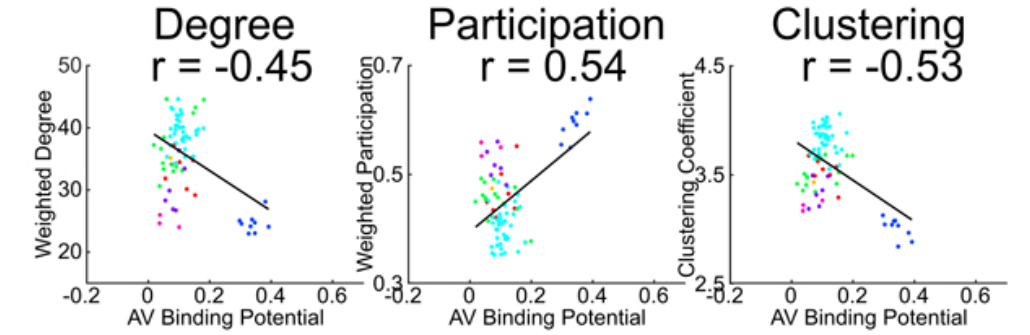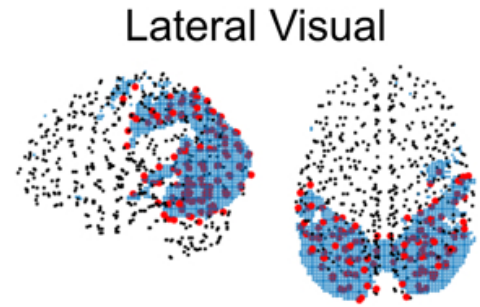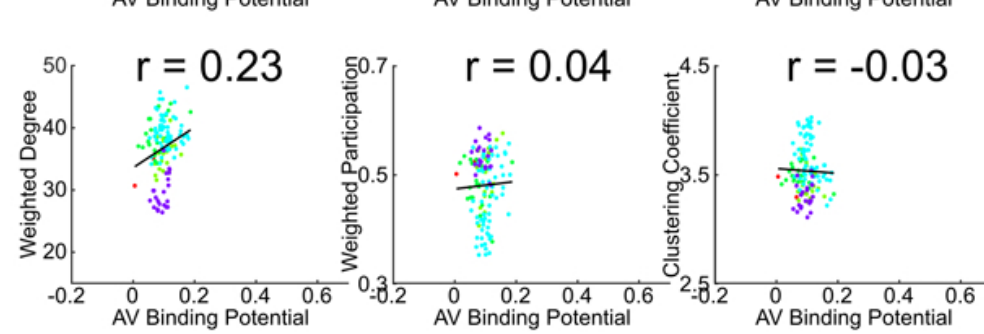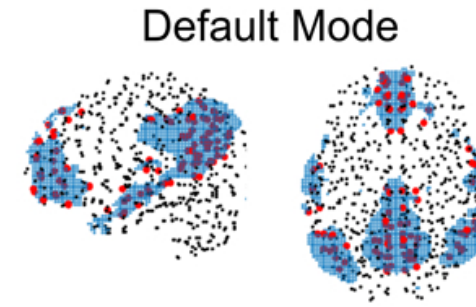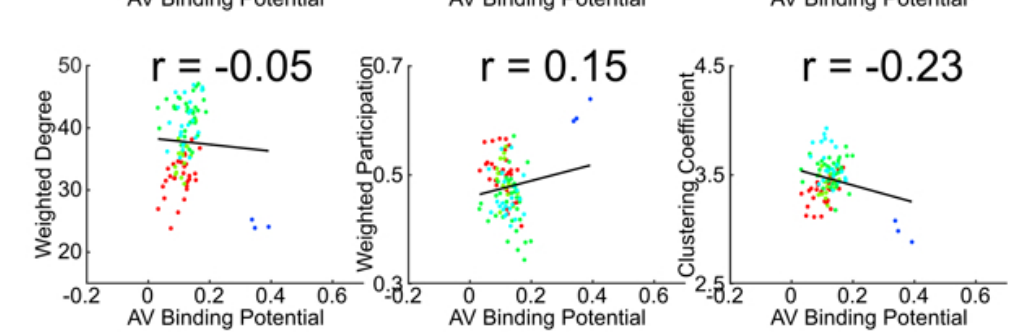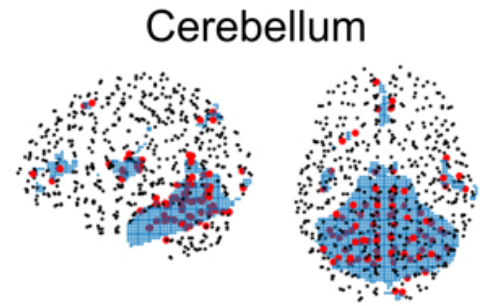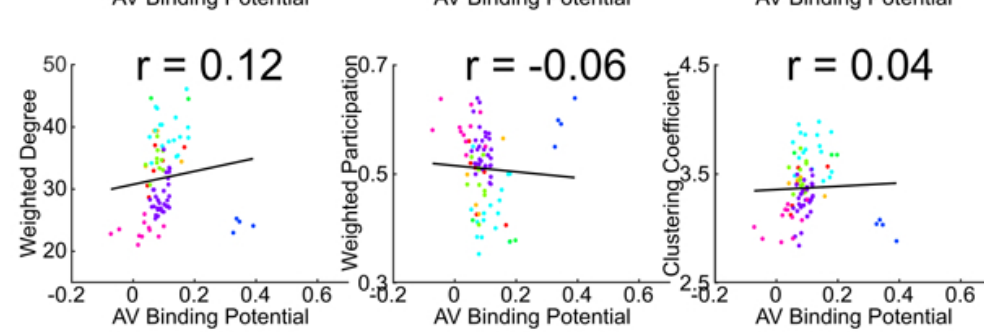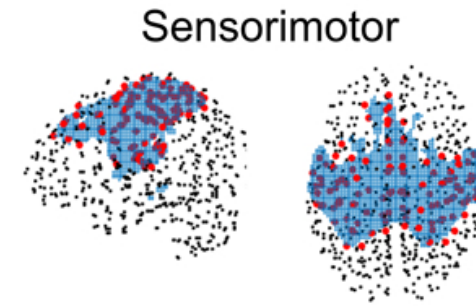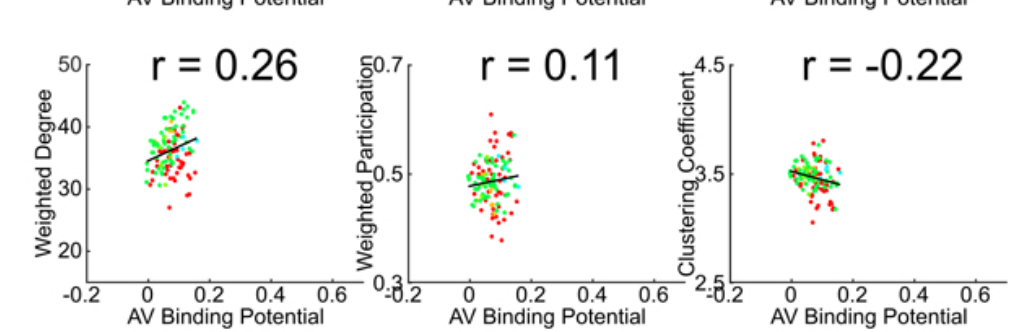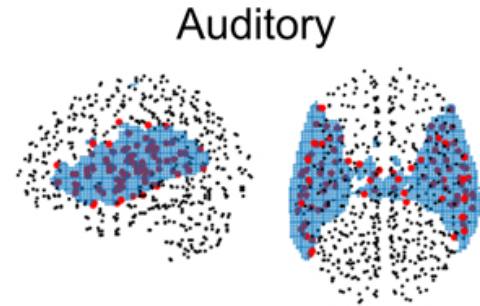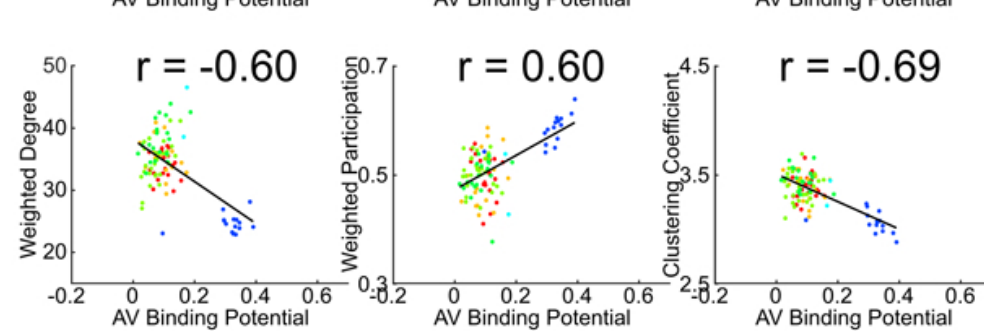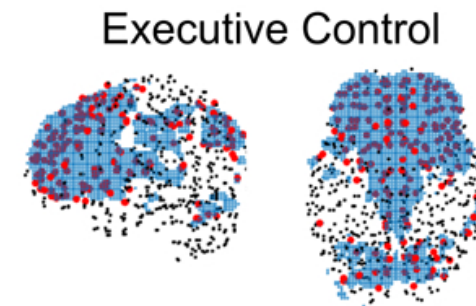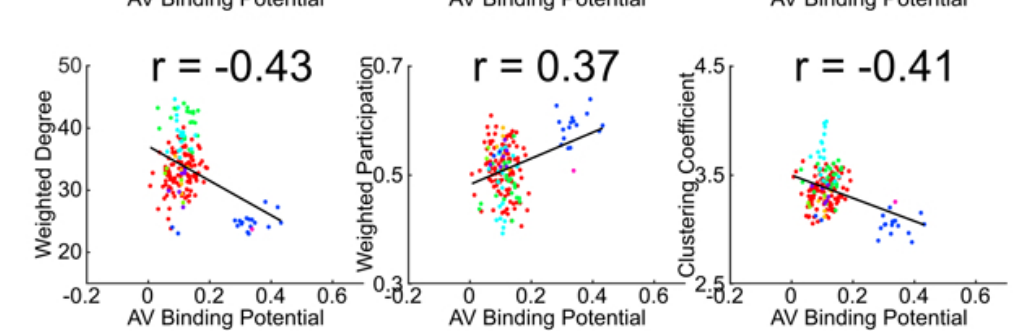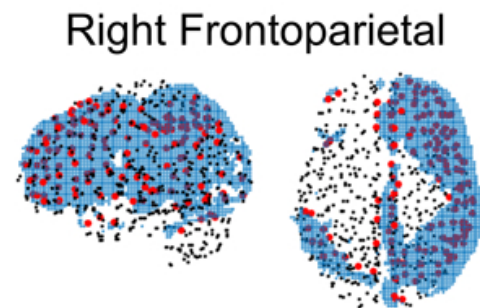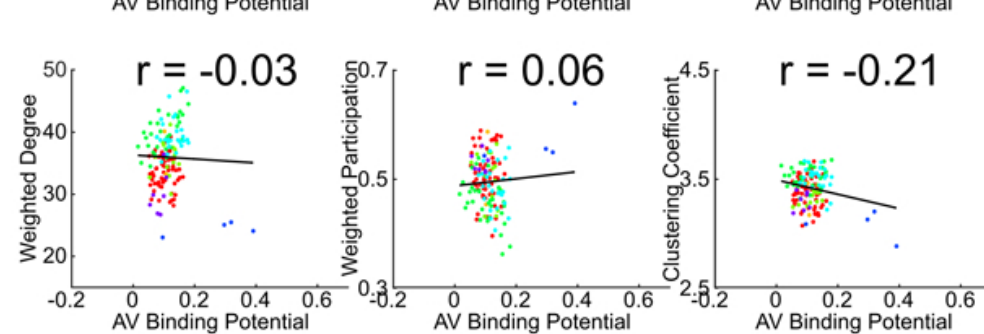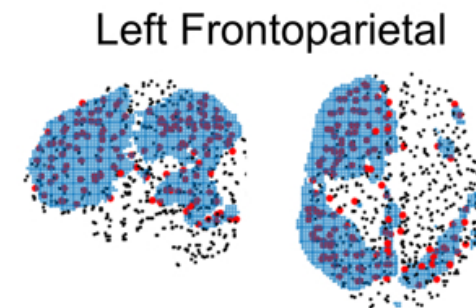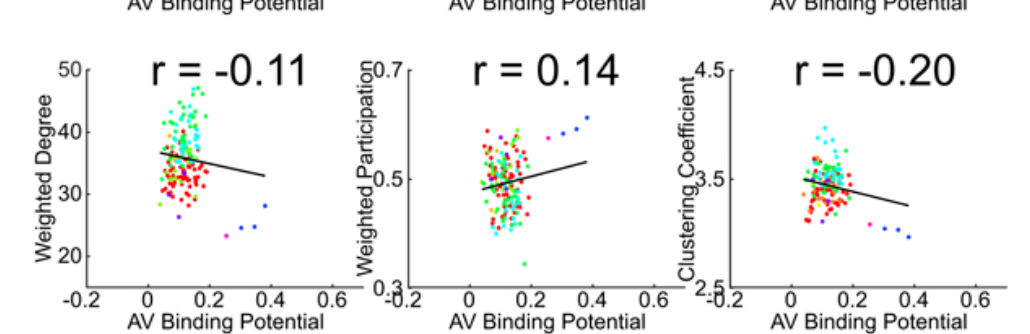

Supplement: Supplementary Figure S4 [file brain-2017-01363-file012_awx347.pdf]
