# Supplementary material for: Tau burden and the functional connectome in Alzheimer’s disease and progressive supranuclear palsy
Source: Brain. 2018 Jan 5;141(2):550–67. doi: 10.1093/brain/awx347 (PMC5837359; doi:10.1093/brain/awx347)
Supplement: Supplementary Figure S1 [file brain-2017-01363-file009_awx347.pdf]

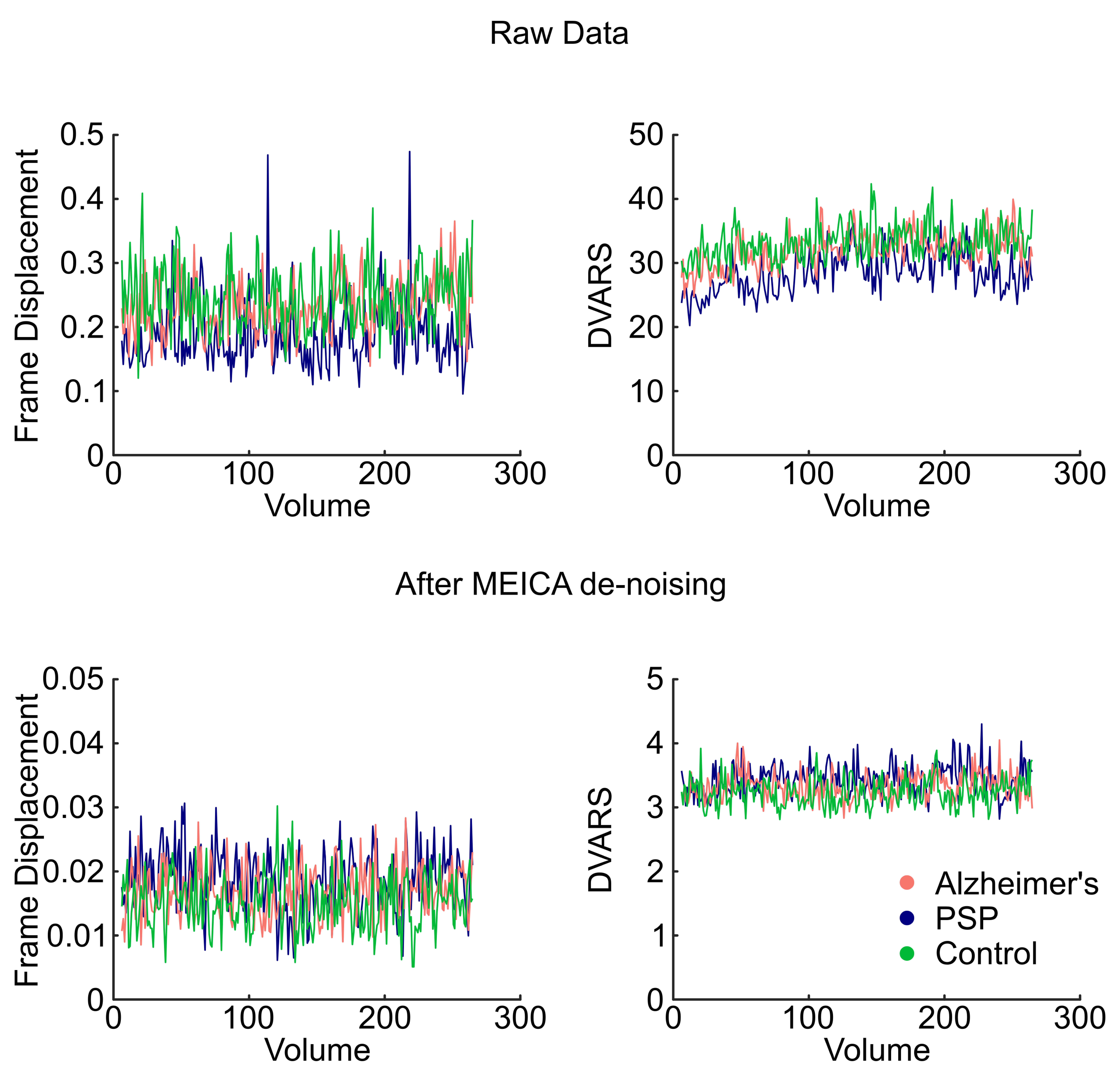

Raw Data
0.5
0.4
0.3
0.2
0.1
0
Frame Displacement
0
100
200
300
Volume
50
40
30
20
10
0
DVARS
0
100
200
300
Volume
After MEICA de-noising
0.05
0.04
0.03
0.02
0.01
0
Frame Displacement
0
100
200
300
Volume
5
4
3
2
1
0
DVARS
0
100
200
300
Volume
Alzheimer's
PSP
Control
