# Supplementary material for: Tau burden and the functional connectome in Alzheimer’s disease and progressive supranuclear palsy
Source: Brain. 2018 Jan 5;141(2):550–67. doi: 10.1093/brain/awx347 (PMC5837359; doi:10.1093/brain/awx347)
Supplement: Supplementary Figure S5 [file brain-2017-01363-file013_awx347.pdf]

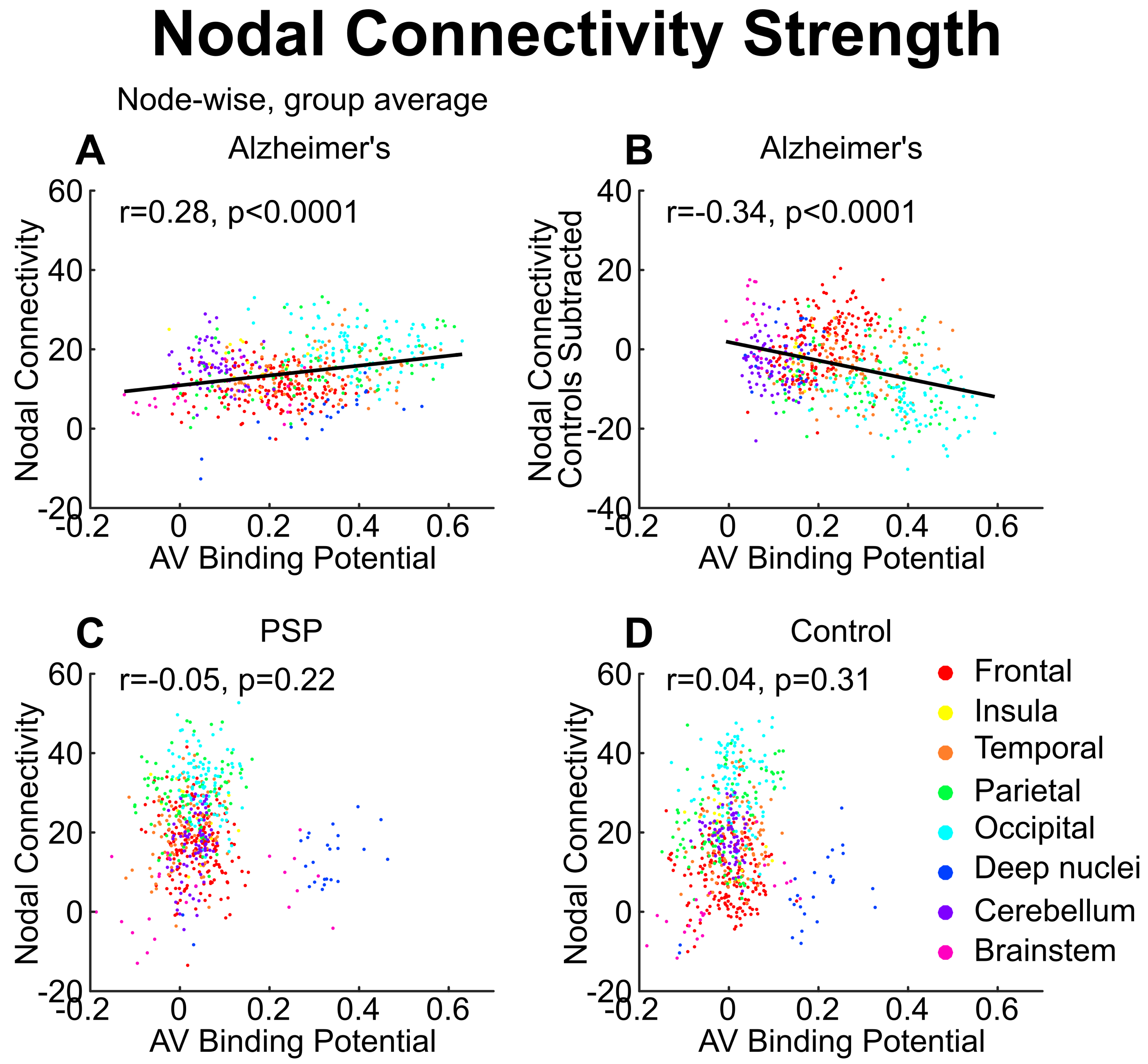
Nodal Connectivity Strength
Node-wise, group average
A
Alzheimer's
r=0.28, p<0.0001
Nodal Connectivity
AV Binding Potential
B
Alzheimer's
r=-0.34, p<0.0001
Nodal Connectivity
Controls Subtracted
AV Binding Potential
C
PSP
r=-0.05, p=0.22
Nodal Connectivity
AV Binding Potential
D
Control
r=0.04, p=0.31
Nodal Connectivity
AV Binding Potential
Frontal
Insula
Temporal
Parietal
Occipital
Deep nuclei
Cerebellum
Brainstem
