# Supplementary material for: Tau burden and the functional connectome in Alzheimer’s disease and progressive supranuclear palsy
Source: Brain. 2018 Jan 5;141(2):550–67. doi: 10.1093/brain/awx347 (PMC5837359; doi:10.1093/brain/awx347)
Supplement: Supplementary Figure S6 [file brain-2017-01363-file014_awx347.pdf]

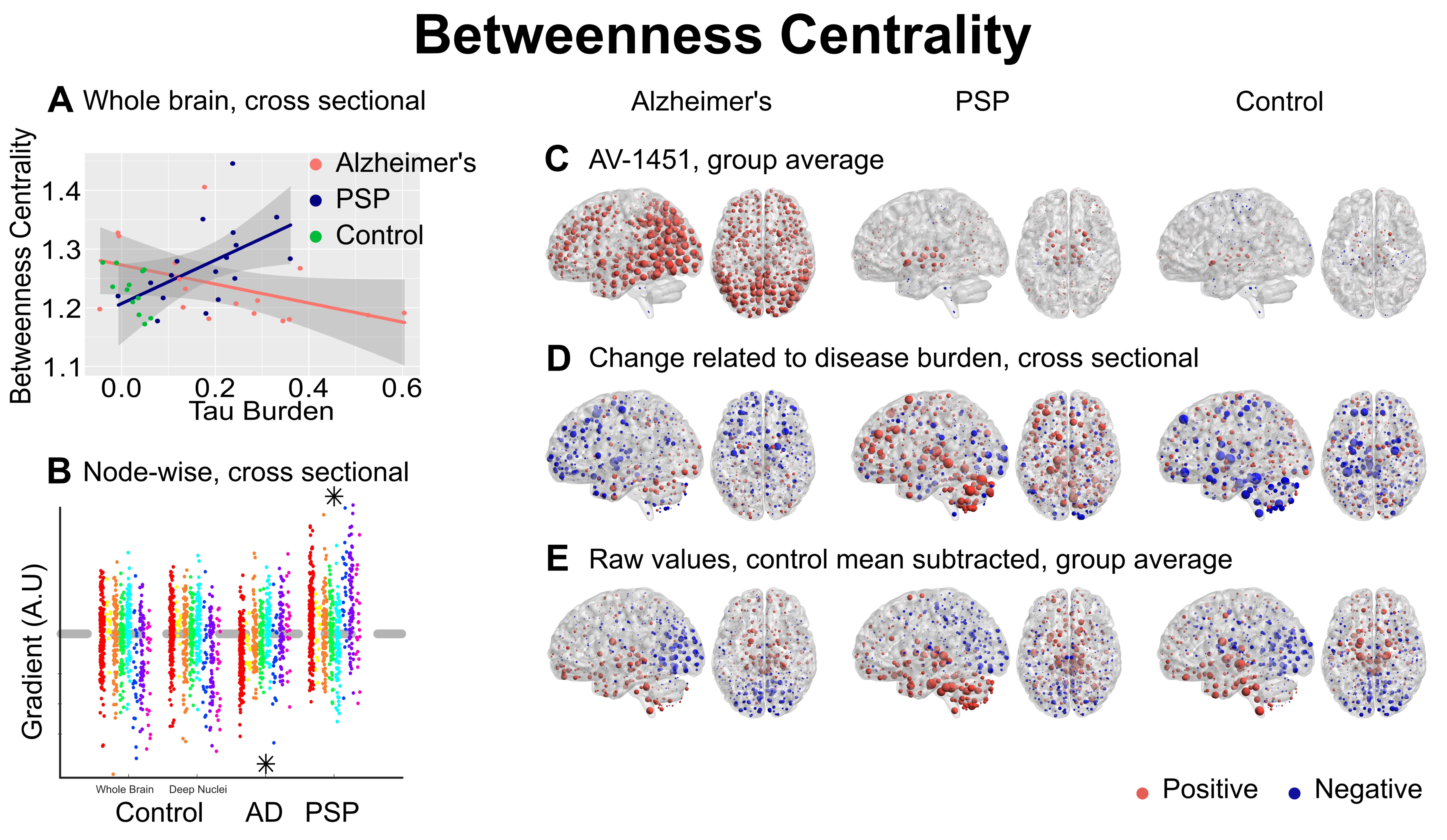

Betweenness Centrality
A Whole brain, cross sectional
Betweenness Centrality
1.4
1.3
1.2
1.1
0.0
0.2
0.4
0.6
Tau Burden
Alzheimer's
PSP
Control
B Node-wise, cross sectional
Gradient (A.U)
Whole Brain
Deep Nuclei
Control
AD
PSP
Alzheimer's
PSP
Control
C AV-1451, group average
D Change related to disease burden, cross sectional
E Raw values, control mean subtracted, group average
Positive
Negative
