# Supplementary material for: Tau burden and the functional connectome in Alzheimer’s disease and progressive supranuclear palsy
Source: Brain. 2018 Jan 5;141(2):550–67. doi: 10.1093/brain/awx347 (PMC5837359; doi:10.1093/brain/awx347)
Supplement: Supplementary Figure S7 [file brain-2017-01363-file015_awx347.pdf]

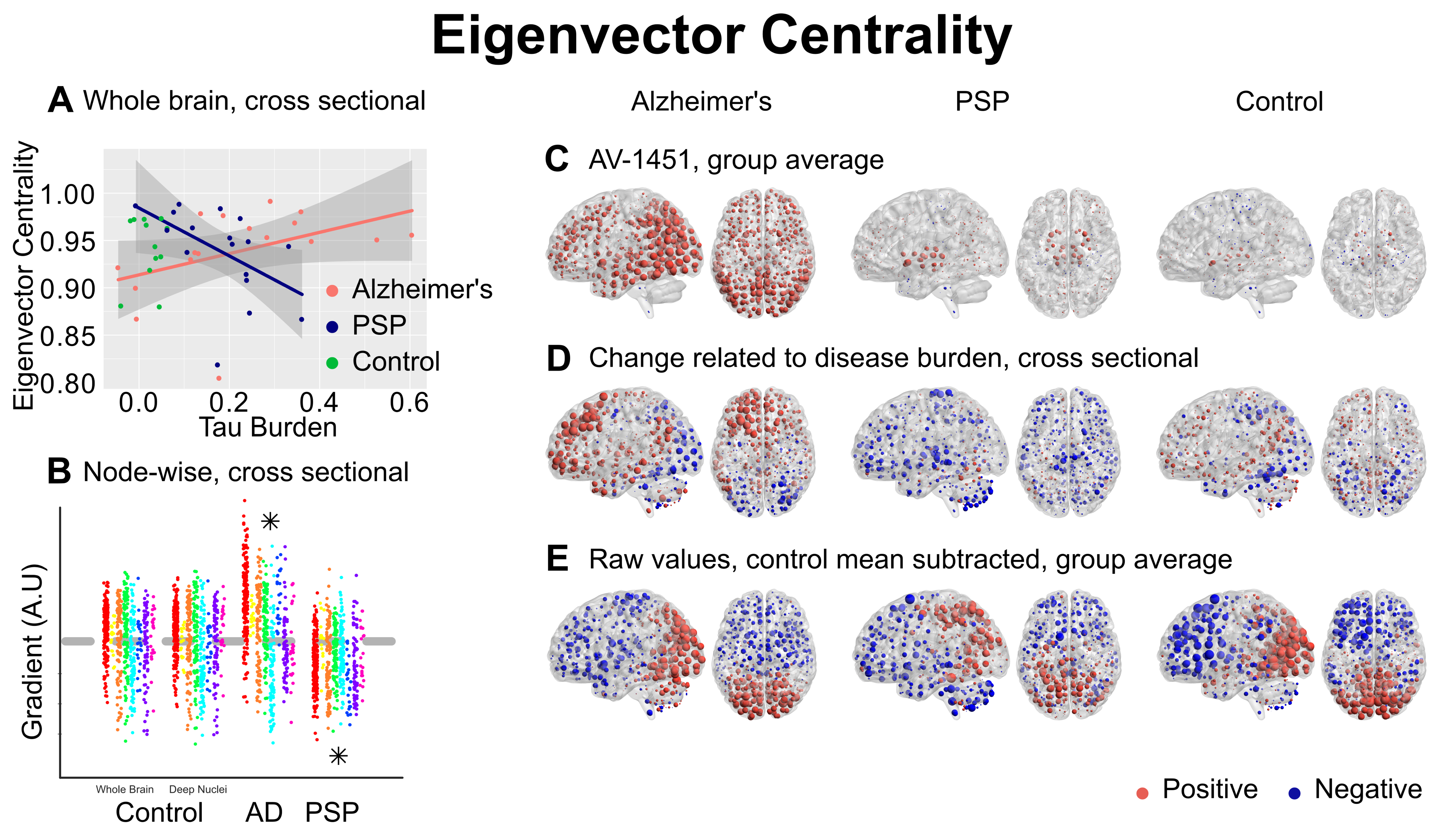
Eigenvector Centrality
A Whole brain, cross sectional
Alzheimer's
PSP
Control
C AV-1451, group average
Eigenvector Centrality
1.00
0.95
0.90
0.85
0.80
0.0
0.2
0.4
0.6
Tau Burden
Alzheimer's
PSP
Control
D Change related to disease burden, cross sectional
B Node-wise, cross sectional
Gradient (A.U)
E Raw values, control mean subtracted, group average
Whole Brain
Deep Nuclei
Control
AD
PSP
Positive
Negative
