# Supplementary material for: Tau burden and the functional connectome in Alzheimer’s disease and progressive supranuclear palsy
Source: Brain. 2018 Jan 5;141(2):550–67. doi: 10.1093/brain/awx347 (PMC5837359; doi:10.1093/brain/awx347)
Supplement: Supplementary Figure Legends [file brain-2017-01363-file018_awx347.pdf]

Supplementary figure 1: Movement parameters as quantified by frame displacement and DVARS (D referring to temporal derivative of timecourses, VARS referring to RMS variance over voxels), before and after MEICA de-noising. No significant differences were demonstrated between groups.

Supplementary figure 2: The relationship between tau Burden and Age for each group. Both methods of calculating tau Burden are shown for the control group. As previously reported, there was a negative trend in Alzheimer's disease, although this was not statistically significant. No such relationship was demonstrated for PSP. Controls also displayed a negative relationship, although this was across a much smaller range of AV-1451 binding.

Supplementary figure 3: PSP comparison of the three graph metrics representing the three principal hypotheses of hub vulnerability. Broken down by intrinsic connectivity network defined from (Smith *et al.*, 2009). The group-averaged graph metric at each node within a network is plotted against [^{18}F-AV-1451 binding potential at that node. The Pearson correlation coefficient is noted in each case.

Supplementary figure 4: Between-subjects analysis of the relationship between global tau burden and each graph metric at a network density of 6%, with the effect of age on tau burden partialled out. A Relative tau Burden of 0 is the age-expected tau Burden within each disease group (i.e. lying on the group trend line in supplementary figure 1). Individuals with lower-than-average tau Burden within their disease group have negative Relative tau Burdens, while those with higher-than-average tau Burden have positive Relative tau Burdens. Moderation analysis for a differential relationship between graph metric and tau burden in the two disease groups (Alzheimer's disease and PSP) was statistically significant for all metrics.

Supplementary figure 5: The relationship between unthresholded nodal connectivity strength and AV-1451 binding. A: Group average for Alzheimer's disease. A statistically significant positive relationship was observed (r = 0.28, $P < 0.0001$). B: The disease-related change in nodal connectivity strength at each node in the Alzheimer's disease group. A statistically significant negative relationship was observed (r = −0.34, $P < 0.0001$). Therefore nodes that were more strongly connected accrued more tau, but the consequence of tau accrual was that those same nodes then lost connectivity. C: Group average for PSP. No statistical relationship was observed

(r = −0.05, P = 0.22). D: Group average for controls. No statistical relationship was observed (r = 0.04, P = 0.31).

Supplementary figure 6: Betweenness centrality. Sub-panels A-E correspond to sub-panels D-H in figure 2.

Supplementary figure 7: Eigenvector centrality. Sub-panels A-E correspond to sub-panels D-H in figure 2.
